# Unique osteogenic profile of bone marrow stem cells stimulated in perfusion bioreactor is Rho‐ROCK‐mediated contractility dependent

**DOI:** 10.1002/btm2.10509

**Published:** 2023-03-17

**Authors:** Shuntaro Yamada, Mohammed A. Yassin, Francesco Torelli, Jan Hansmann, Jeremy B. A. Green, Thomas Schwarz, Kamal Mustafa

**Affiliations:** ^1^ Center of Translational Oral Research (TOR)‐Tissue Engineering Group, Department of Clinical Dentistry, Faculty of Medicine University of Bergen Norway; ^2^ Translational Center Regenerative Therapies Fraunhofer Institute for Silicate Research ISC Würzburg Germany; ^3^ Chair of Tissue Engineering and Regenerative Medicine University Hospital Würzburg Würzburg Germany; ^4^ Department of Electrical Engineering University of Applied Sciences Würzburg‐Schweinfurt Schweinfurt Germany; ^5^ Centre for Craniofacial & Regenerative Biology, Faculty of Dentistry, Oral & Craniofacial Sciences King's College London UK

**Keywords:** actomyosin contraction, bioreactor, bone tissue engineering, fluid shear stress, osteogenic differentiation, Rho GTPase signaling

## Abstract

The fate determination of bone marrow mesenchymal stem/stromal cells (BMSC) is tightly regulated by mechanical cues, including fluid shear stress. Knowledge of mechanobiology in 2D culture has allowed researchers in bone tissue engineering to develop 3D dynamic culture systems with the potential for clinical translation in which the fate and growth of BMSC are mechanically controlled. However, due to the complexity of 3D dynamic cell culture compared to the 2D counterpart, the mechanisms of cell regulation in the dynamic environment remain relatively undescribed. In the present study, we analyzed the cytoskeletal modulation and osteogenic profiles of BMSC under fluid stimuli in a 3D culture condition using a perfusion bioreactor. BMSC subjected to fluid shear stress (mean 1.56 mPa) showed increased actomyosin contractility, accompanied by the upregulation of mechanoreceptors, focal adhesions, and Rho GTPase‐mediated signaling molecules. Osteogenic gene expression profiling revealed that fluid shear stress promoted the expression of osteogenic markers differently from chemically induced osteogenesis. Osteogenic marker mRNA expression, type 1 collagen formation, ALP activity, and mineralization were promoted in the dynamic condition, even in the absence of chemical supplementation. The inhibition of cell contractility under flow by Rhosin chloride, Y27632, MLCK inhibitor peptide‐18, or Blebbistatin revealed that actomyosin contractility was required for maintaining the proliferative status and mechanically induced osteogenic differentiation in the dynamic culture. The study highlights the cytoskeletal response and unique osteogenic profile of BMSC in this type of dynamic cell culture, stepping toward the clinical translation of mechanically stimulated BMCS for bone regeneration.

## 1 INTRODUCTION

The fate and growth of bone marrow mesenchymal stem/stromal cells (BMSC) are tightly regulated by mechano‐environmental factors.^1^, ^2^ The forces of load and torsion applied to bone are converted to shear forces by altered interstitial fluid flow in bone marrow, which is exerted on the resident cells, including mechanosensitive BMSC.^3^ For example, hydrostatic pressure, fluid shear stress, and altered rheological properties of BMSC niche regulate their fate into osteoblast, adipocyte, chondrocyte, or stromal cell, balancing bone homeostasis and remodeling.^3^, ^4^ Physical activity increases fluid shear stress in bone marrow, which promotes bone remodeling by energizing osteoblasts and their progenitors, whereas a disbalanced mechano‐environment by physiopathological conditions such as aging, diseases (i.e., osteoporosis), and disuse favors adipogenesis.^3^, ^5^, ^6^, ^7^, ^8^, ^9^

The osteogenic nature of BMSC under fluid shear and its plausible mechanism have been predominantly described by conventional monolayer cell culture experiments. In osteoinductive medium (i.e., in the presence of dexamethasone, beta‐glycerophosphate, and ascorbic acid), BMSC preferably respond to fluid shear stress as small as 0.01 Pa up to 2 Pa by upregulating osteogenic markers such as Runt‐related transcription factor 2 (Runx2), Osteopontin (Opn), Bone morphogenetic protein 2 (Bmp2), alkaline phosphatase (ALP), collagen type 1 (Col1), and Osteocalcin (Ocn) accompanied by increased mineralization and extracellular matrix formation.^10^, ^11^, ^12^, ^13^, ^14^, ^15^, ^16^, ^17^, ^18^, ^19^ BMSC subjected to fluid flow undergo dynamic cytoskeletal rearrangement moderated by integrins, focal adhesion, MAPK/ERK signaling, Rho‐ and Hippo‐YAP/TAZ signaling, which are tightly linked to osteogenic differentiation.^10^, ^12^, ^13^, ^20^, ^21^, ^22^ The evidence of flow‐induced osteogenic differentiation in the absence of the osteoinductive medium is rather limited. Approximately 1 Pa shear stress was reported to increase the enzymic activity of ALP and the expression of osterix (Osx), Opn, and Col1, but whether these cellular responses can be considered as osteogenic differentiation is debatable.^21^, ^23^, ^24^

The knowledge gained from the field of mechanobiology has been transferred into tissue engineering for bone regeneration where three‐dimensional (3D) scaffolds are combined with BMSC to create transplantable constructs. With 3D scaffolds, gas, nutrient, and waste transport to/from the loaded cells has mostly relied on passive diffusion in static culture, causing the heterogeneity of cell growth and regenerative capacity.^25^, ^26^ Therefore, high expectations have been placed on the use of perfusion bioreactor systems to improve cell production. The rationale of applying bioreactors in bone tissue engineering is threefold: to uniform nutrient and gas distribution within 3D constructs to ensure the homogeneity of cell distribution and their functionality among the constructs, to mimic the mechanical environment of bone and bone marrow to stimulate osteogenic properties of the cells, and to automate cell production process to reduce the risk of human‐error and improve cost‐effectiveness.^27^ Similar to the 2D observations, studies using the various prototypes of bioreactors, mostly in the presence of the osteoinductive medium, reported the preferable effects of 3D perfusion culture on osteogenic differentiation.^28^, ^29^, ^30^, ^31^, ^32^, ^33^, ^34^, ^35^ Notably, the concept was proven by a study that BMSC co‐cultured with vascular endothelial cells in the osteoinductive medium that was preconditioned in a perfusion bioreactor improved bone formation and visualization after transplantation into calvaria bone defects.^29^ We previously reported that the osteogenesis of BMSC could be induced mechanically by the sub‐physiological level of flow, even in the absence of the osteoinductive medium, showing the upregulated expression of osteogenic markers and the improved functionality of the cells including matrix formation and mineralization in a perfusion bioreactor.^36^ Interestingly, BMSC in a 3D environment are reportedly more perceptive to shear stress than that in a 2D environment, but the cellular mechanisms of mechanically stimulated osteogenesis in a 3D dynamic culture remain underdocumented.^37^ To translate the technology into clinical application, in depth understanding of cellular response is necessary. Therefore, in the present study, we aimed at elucidating mechanically induced osteogenesis by fluid flow on 3D porous scaffolds by focusing on signaling in cytoskeletal rearrangement and osteogenic profiling in depth. The dynamic cell culture was performed using a custom‐designed bioreactor with experimental parameters disclosed, and flow characteristics was computationally estimated to correlate the magnitude of shear stress with cellular behaviors.

## 2 MATERIALS AND METHODS

### 2.1 BMSC isolation and cell culture

The study was approved by the Norwegian Animal Research Authority (local approval number 20146866) and conducted according to the European Convention for the Protection of Vertebrates used for scientific purposes. BMSC were isolated from femurs of male Lewis rats euthanized by lethal doses of carbon dioxide and cervical dislocation. The cells were maintained for both static and dynamic culture in α‐minimum essential medium (α‐MEM: 22571; Gibco™, USA) supplemented with 1% penicillin and streptomycin (SV30010; HyClone, USA) and 10% fetal bovine serum (FBS: 10270‐106; Gibco™, USA) at 37°C in 5% CO_2_. The expression of putative stem cell markers and the capacity of multilineage differentiation were previously verified.^36^ BMSC from the third to fifth passages were used in the study. Osteoinductive medium (OM) used as a positive control for osteogenic differentiation consisted of α‐MEM supplemented with 1% penicillin and streptomycin, 10% FBS, 10 nM dexamethasone (D4902; Sigma, USA), 10 mM beta‐glycerophosphate (G9422; Sigma, USA), and 173 μM l‐ascorbic acid (A8960; Sigma, USA).

### 2.2 Preparation of 3D porous scaffolds of poly(l‐lactide‐*co*‐trimethylene carbonate)

3D porous scaffolds with a diameter of 12 mm and a thickness of 1.2 mm were fabricated by a solvent‐casting/salt‐leaching technique as described previously.^38^ Briefly, synthetic polymers, poly(l‐lactide‐*co*‐trimethylene carbonate) 70:30 (RESOMER® LT 706S; Evonik, Germany), were dissolved in chloroform and mixed with sodium chloride (NaCl) at a weight ratio of 1:10. After the slow evaporation of chloroform, the scaffolds were thoroughly washed to remove NaCl particles. Scaffolds were sterilized by 75% ethanol and UV irradiation prior to cell seeding. A total of 250,000 cells were seeded per scaffold in 48 well plates.

### 2.3 Bioreactor system and 3D dynamic cell culture under perfusion

**FIGURE 1:**
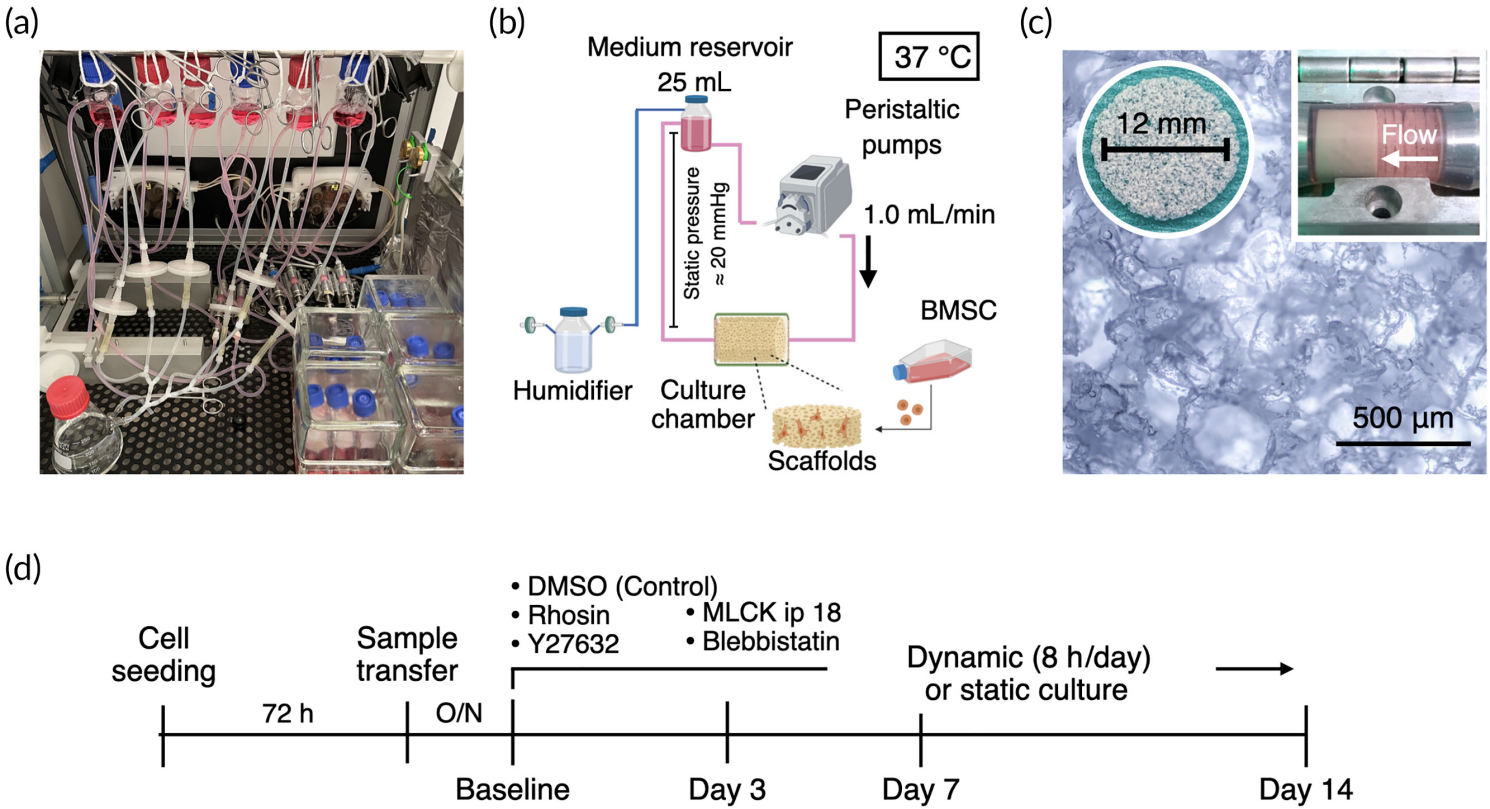
Experimental design and perfusion bioreactor. (a) The bioreactor used for 3D dynamic culture in the present study consists of integrated incubator and perfusion systems. (b) Schematic illustration of experimental setting including environmental factors in the dynamic cell culture. (c) Five cell‐laden porous scaffolds of poly(l‐lactide‐*co*‐trimethylene carbonate) were placed in the sample chambers where 25 mL of growth medium was perfused at 1.0 mL/min. (d) Experimental timeline.

A custom‐designed GMP‐complied laminar flow perfusion bioreactor was utilized in the study. It consisted of an integrated incubator system with environmental sensors (i.e., temperature, gas concentration, pressure: details previously described^39^) and peristaltic pumps (Figure 1a). In the bioreactor, 25 mL of the growth medium was perfused, and the medium was refreshed twice a week unless otherwise mentioned (Figure 1b). The medium reservoirs were connected to ventilation filters via humidifiers to allow for gas exchange while minimizing medium evaporation. Approximately 20 mmHg of hydrostatic pressure was applied to sample chambers where five cell‐laden scaffolds were placed in order to prevent air bubble formation during the dynamic cell culture (Figure 1c). Optimal flow rate for the cells was preliminarily determined at 1.0 mL/min.^39^

The experimental flow was given in Figure 1d. Briefly, 72 h after cell seeding on the scaffolds, five cell‐laden scaffolds were transferred into the sample chamber in the bioreactor. The following day was defined as the baseline when dynamic cell culture has begun. Dynamic cell culture continued for 8 h a day for 14 days. As a control, five cell‐laden scaffolds were stacked and placed in a flask with 25 mL medium in the incubator space of the bioreactor to equalize the cell‐to‐medium ratio and environmental fluctuation. The samples were collected on Days 3, 7, and 14 for analyses, and the experiment was independently repeated three times unless otherwise mentioned.

### 2.4 Computational fluid dynamics simulation

For flow characterization during dynamic cell culture, an in silico modeling was performed as previously described.^39^ Briefly, the geometry of the scaffolds was obtained by microcomputed tomography (microCT: SkyScan 1172;Bruker‐MicroCT, Kontich, Belgium) using 40 kV and 250 mA at 10 μm spatial resolution. The geometry was then imported in *.stl file* as a solid object using COMSOL Multiphysics version 6.0 (COMSOL AB, Sweden). Scaffold geometries were placed in a digitally reproduced sample chamber. From the inlet, fully developed flow of 1.0 mL/min was prescribed. At the outlet, pressure at 8.6 Pa was prescribed as a boundary condition, which was defined based on pressure drop over the scaffolds in a macro model as previously proposed.^39^, ^40^ Incompressible Newtonian fluid (described by Navier–Stokes equations) with a dynamic viscosity of 0.6922 mPa*s and density of 993.37 kg/m^3^ was defined, which was governed by the Navier–Stokes equation and the continuity equation as follows:

$$\rho \left(u.\nabla \right)u=\nabla .\left[-\mathrm{pI}+K\right]+F$$

ρ∇.u=0

$$K=\mu \left(\nabla u+{\left(\nabla u\right)}^{T}\right)$$

where 𝜌, 𝑢, ∇, 𝑝, 𝐼, 𝐾, 𝐹, and *μ* are the fluid density, the fluid velocity, the divergence operator, the pressure, the identity tensor, the stress tensor, the body force per unit volume, and the dynamic viscosity of the fluid, respectively.

Nonslip boundary conditions were implemented at the solid walls.

### 2.5 Pharmacological modulation of actomyosin contractility

To investigate the role of cytoskeletal rearrangement in BMSC growth and differentiation under flow, four inhibitors and two enhancers of actomyosin contractility were applied.

For the inhibition, 20 μM Rhosin chloride (Rhosin: 5003; Biotechne, UK), 10 μM Y27632 dihydrochloride (Y27632: 1254; Biotechne, UK), 1 μM MLCK inhibitor peptide 18 (MLCK ip 18: HY‐P1029; MedChemExpress, USA), or 10 μM Blebbistatin (203390; Sigma‐Aldrich, USA) was added to the culture medium during the dynamic cell culture to inhibit Rho GTPase, Rho‐associated coiled‐coil containing protein kinase (ROCK), myosin light chain kinase (MLCK), and myosin II, respectively (Figure S1A). The optimal concentrations of each inhibitor were determined preliminarily as the concentrations that mitigated actomyosin contractility without deteriorating cell viability and growth for 14 days (Figure S1). It was also confirmed that these inhibitors did not significantly influence on Runx2 expression in the static condition without the osteoinductive stimuli. Narciclasine (Narc: HY‐16563; MedChemExpress, USA) and Calyculin A (CalA: sc‐24000; Santa Cruz Biotechnology, USA) were used to activate Rho‐ROCK signaling and to induce actomyosin contraction via the inhibition of myosin light chain phosphatase, respectively (Figure S2A,B). Dimethyl sulfoxide (DMSO) was used as a solvent and acted as a control. The culture media with inhibitors were refreshed every 2 days for 14 days.

### 2.6 Reverse transcription‐quantitative polymerase chain reaction and gene expression array

Samples for gene expression assay were collected on Days 7 and 14. After being immediately snap‐frozen in liquid nitrogen, the samples were stored at −80°C. Total RNA was extracted using a Maxwell® 16 Cell LEV Total RNA Purification Kit (AS1280; Promega, USA) in accordance with the manufacturer's protocol. Reverse transcription was undertaken using a High‐Capacity cDNA reverse Transcription Kit (4368813; Applied Biosystems, USA). RT‐qPCR was performed with the StepOne™ real‐time PCR system (4376357; Applied Biosystems) with TaqMan® Universal Master Mix (4352042; Applied Biosystems). The amplification was performed as follows: initial activation of polymerase at 95°C for 20 s followed by 40 cycles of PCR, at 95°C for 1 s (denature) and 60°C for 20 s (anneal and extend).

The tailored panels of gene expression assay for cytoskeletal rearrangement and osteogenesis were designed with reference to the predetermined TaqMan® gene expression arrays for focal adhesion (4413255‐RPMFWWX; Applied Biosystems), cytoskeleton regulators (4413255‐RPPRJ2T; Applied Biosystems), and osteogenesis (4413255‐RPZTD3T; Applied Biosystems). A set of primers used in the study is listed in Tables [Link], [Link]. Relative expression of each mRNA was calculated with the ΔΔCt method.^41^

### 2.7 Gene set enrichment analysis

A list of differentially expressed genes (DEGs) identified through statistical analysis was further analyzed using the R package gprofiler2 and the STRING database version 11.5. Functional enrichment of the DEGs was evaluated using the open‐source software Cytoscape version 3.9.1 by referencing the Reactome Pathways and UniProt databases.

### 2.8 Cytoskeleton antibody microarray

To capture the overview of cytoskeletal rearrangement, ELISA‐based antibody microarray was performed using Cytoskeleton Array kit (PCP141; Full Moon BioSystem, USA) according to the manufacturer's protocol. Briefly, samples were collected on Day 3, and protein extraction was performed by vigorous vortexing with lysis beads in the extraction buffer. Due to low protein yield, cell lysate obtained from three independent experiments was pooled. The protein samples were labeled in biotin dissolved in dimethylformamide in the labeling buffer. The biotinylated samples were then incubated with the antibody microarray slides followed by detection with Cy3‐Streptavidin solution. The slides were scanned by a GenePix® Microarray Scanner (Molecular Devices, USA) and quantified using GenePix® ProMicroarray Image Analysis Software.

### 2.9 ROCK enzymic activity assay

The enzymic activity of ROCK was measured on Days 3 and 7 by using ROCK Activity Assay Kit (ab211175; Abcam, USA) according to the manufacturer's protocol. Cell lysate was obtained in mammalian cell lysis buffer (ab179835; Abcam) with protease and phosphatase inhibitor cocktail (ab201119; Abcam). The cell lysate was added to wells where myosin phosphate target subunit 1 (MYPT1) was coated. Kinase reaction was initiated by adding 10 mM dithiothreitol (DTT) and 2 mM adenosine triphosphate (ATP). The wells were then incubated for 60 min at room temperature to phosphorylate MYPT1. Subsequently, the wells were incubated with anti‐phospho‐MYPT1 (Thr696) for 60 min at room temperature followed by secondary incubation with HRP‐conjugated antibody for another 60 min at room temperature. The optical density of the HRP substrates was measured at 450 nm using a Varioskan™ LUX multimode microplate reader (VLBL00D0; Thermo Scientific, Finland).

### 2.10 Immunofluorescence and confocal microscopy

For immunofluorescence, samples were fixed in 4% paraformaldehyde (PFA) for 15 min at room temperature except for collagen staining. To detect phosphorylated proteins, fixation was undertaken in the presence of protease and phosphatase inhibitor (ab201119; Abcam, UK). The samples were then permeabilized in 0.1% Triton X‐100 in PBS for 15 min at room temperature. For collagen staining, samples were fixed and permeabilized in ice‐cold methanol for 5 min at −20°C. The samples were then incubated in a blocking buffer consisting of 10% normal goat serum (NGS: ab7481; Abcam, USA) in 0.1% Tween‐20 in PBS (PBSTw) for 60 min at room temperature. After blocking, the samples were incubated with the following primary antibodies in PBSTw at 4°C overnight: rabbit anti‐non‐muscle myosin 2A antibody (NM2A: 1:200, 909801; BioLegend, USA), mouse anti‐non‐muscle myosin 2B antibody (NM2B: 1:200, GTX634160; GeneTex, USA), mouse anti‐phospho‐MYL9 (Ser19) antibody (pMLC2: 1:250, MA5‐15163; Invitrogen, USA), and mouse anti‐collagen type 1 antibody (Col1: 1:500, MA1‐26771; Invitrogen). Subsequently, the samples were incubated with secondary antibodies, goat anti‐rabbit antibody, Alexa Fluor 546 (1:500, A11010; Invitrogen) and/or goat anti‐mouse antibody, Alexa Fluor 635 (1:500; A31575; Invitrogen) for 1 h at room temperature simultaneously with 4′,6‐diamidino‐2‐phenylindole (DAPI: 1:2500, D9542; Sigma‐Aldrich, USA) and Phalloidin Alexa488 (1:500, A12379; Invitrogen) for nuclear staining and filamentous actin staining, respectively.

For image acquisition, the samples were placed on chambered coverslips (80287, ibidi, Germany) and mounted in ProLong™ Gold antifade reagent (P36939; Invitrogen). Z‐Stack images were acquired by a confocal microscope (TCS SP8; Leica, Germany) equipped with a 40× water immersion objective lens. The images were processed and analyzed with Fiji/ImageJ.^42^ All images are presented as z‐stack projection images of 100 μm thickness.

### 2.11 Quantification of double‐strand DNA

To assess cell proliferation, double‐strand DNA (dsDNA) quantification was performed using Quant‐iT PicoGreen dsDNA Assay Kit (P7589; Invitrogen) according to the manufacturer's protocol. Cell lysate was obtained by repeated freeze–thaw cycle in 0.1% Triton X‐100 in Milli‐Q® water. The intensity of fluorescence was measured at Ex/Em = 480/520 nm using the microplate reader.

### 2.12 5‐Ethynyl‐2′‐deoxyuridine incorporation assay

For EdU assay, 30 μM EdU was added to the growth medium on Day 3 in the static and dynamic culture. Twenty‐four hours after EdU administration, the samples were fixed in ice‐cold methanol for 5 min at −20°C. Incorporated EdU to the nuclei was detected using Click‐iT™ EdU Cell Proliferation Kit for Imaging, Alexa Fluor™ 488 dye (C10337; Invitrogen) according to the manufacturer's protocol and visualized by the Leica SP8 confocal microscope.

### 2.13 ALP staining

The samples collected on Day 7 were placidly fixed in 4% PFA for 2 min at room temperature. The samples were then incubated with BCIP®/NBT solusion (B5655; Sigma‐Aldrich) for 30 min at room temperature. For quantification, the substrate was extracted by incubating with 100 mM cetylpyridium chloride overnight at room temperature. Absorbance was measured at 540 nm using the microplate reader.

### 2.14 Alizarin Red S staining

Samples collected on Day 14 were fixed in 4% PFA for 40 min and washed gently in Milli‐Q® water. Mineralized nodes were stained by 0.1% Alizarin Red S (A5533; Sigma‐Aldrich) for 20 min followed by washing six times in Milli‐Q® water. For quantification, the dye was extracted with 100 mM cetylpyridium chloride overnight at room temperature. Absorbance was measured at 540 nm using the microplate reader.

### 2.15 Statistics

All data are represented as mean ± s.e.m unless otherwise specified. Statistical analyses were performed by using Prism 9 (Dotmatics, USA). For comparison between the static and dynamic culture conditions, Student's *t*‐test was performed. For multiple comparisons, the data were evaluated by ANOVA followed by Dunnett's multiple comparisons test. In the cytoskeletal inhibition experiments, the mean of the chemically treated groups was compared to the control (i.e., DMSO) group only. A *p* value <0.05 was considered to be statistically significant.

## 3 RESULTS

### 3.1 CFD assessment of fluid velocity and shear stress in the bioreactor

**FIGURE 2:**
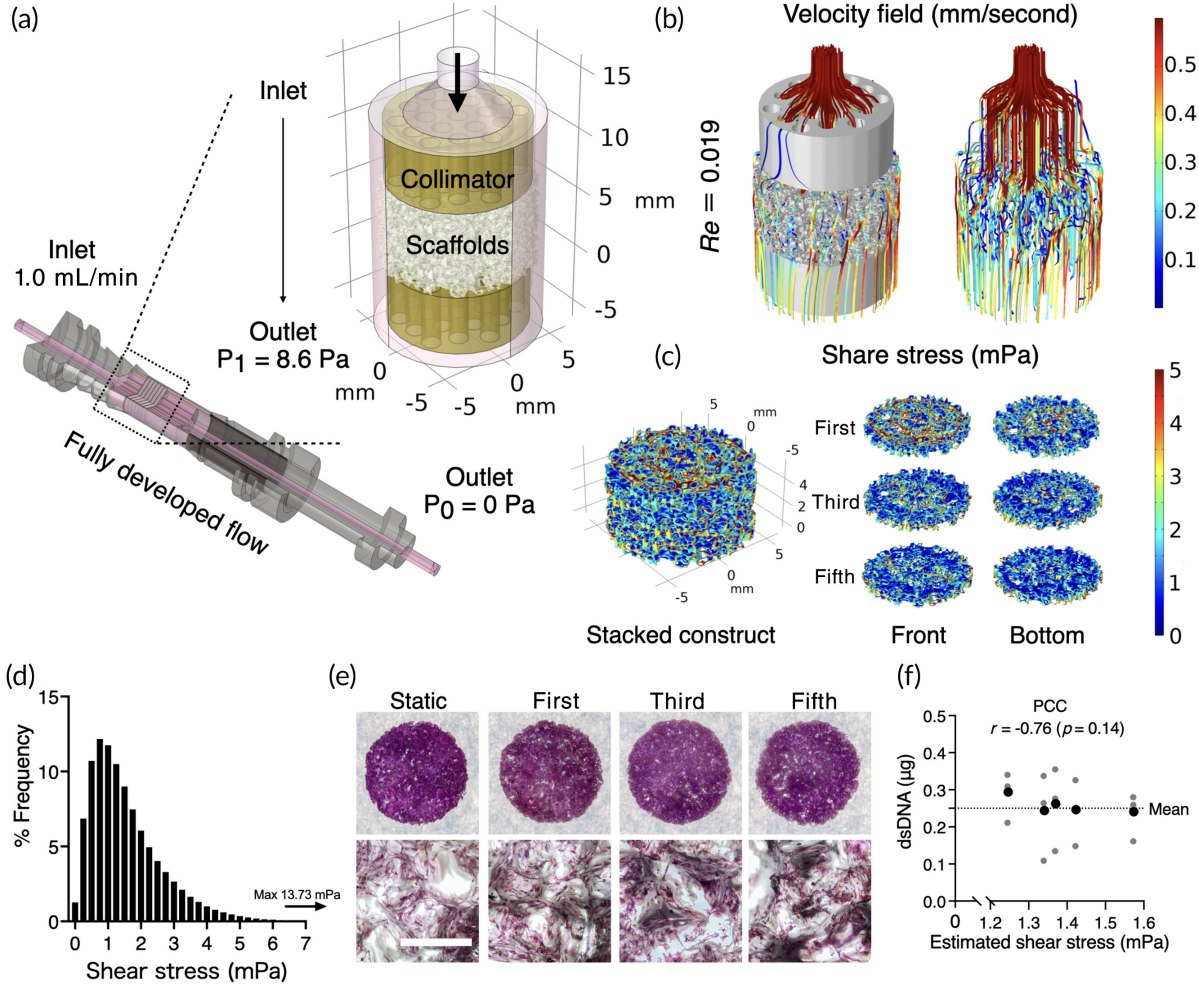
Computational fluid dynamics (CFD) for fluid shear stress estimation (a) The scaffold geometry obtained by a micro computed tomography was virtually placed in the scaffold chambers where fully developed flow at 1.0 mL/min was provided at the inlet. At the outlet of the chamber, atmospheric pressure was prescribed (P0), which determined pressure at the end of collimators on the outlet side at 8.6 Pa (P1). (b) Fluid paths were simulated evenly among the scaffolds with Reynolds number at 0.019, indicating the flow was laminar. (c) Simulation of fluid shear stress on the surfaces of first, third, and fifth scaffolds in the stack showed local shear distribution. (d) Frequent distribution of fluid shear stress ranged from 1.5e‐008 to 13.73 mPa. (e) Crystal violet staining showed actual cell distribution among the scaffold stacks. (f) Pearson correlation coefficient (PCC) indicated no correlation between the estimated shear stress and harvested double‐strand DNA (dsDNA) from the scaffolds at different position after 7 days of perfusion culture. Mean of harvested dsDNA (in black) were calculated from three independent samples (in gray). Scale bar = 1 mm.

Prior to perfusion cell culture, in silico CFD simulation was conducted to describe the distribution and magnitude of shear stress within the stack of the porous scaffolds based on micro‐CT imaging of the scaffold structure (Figure 2a). A flow rate was preliminarily determined by evaluating cell growth, and 1.0 mL/min was considered optimal for the study. With the flow rate, fluid paths were evenly distributed among the stacks through the collimators, and estimated velocity field ranged from 0.1 to 0.5 mm/second (Figure 2b). Computed shear stress on the surfaces showed local variation within the stack, and the highest magnitude was estimated on the surfaces the scaffold closest to the inlet (Figure 2c). Estimated shear stress ranged from 1.5e‐008 to 13.73 mPa due to the heterogeneous nature of complex 3D geometry (Figure 2d). The mean and median values of estimated shear stress were 1.56 and 1.29 mPa, respectively, and over 90% of the surfaces was subjected to a range of shear stress between 0.5 and 5 mPa. DNA staining with crystal violet showed no visible difference in cell distribution between successive scaffolds in the stack (Figure 2e). Pearson correlation coefficient (PCC) did not find the correlation between the amount of dsDNA harvested and the estimated median shear stress in each scaffold, indicating that cell behaviors in the dynamic condition would be similar throughout the stack (Figure 2f).

### 3.2 Fluid shear stress‐induced dynamic cytoskeletal rearrangement and contractility in the 3D system

**FIGURE 3:**
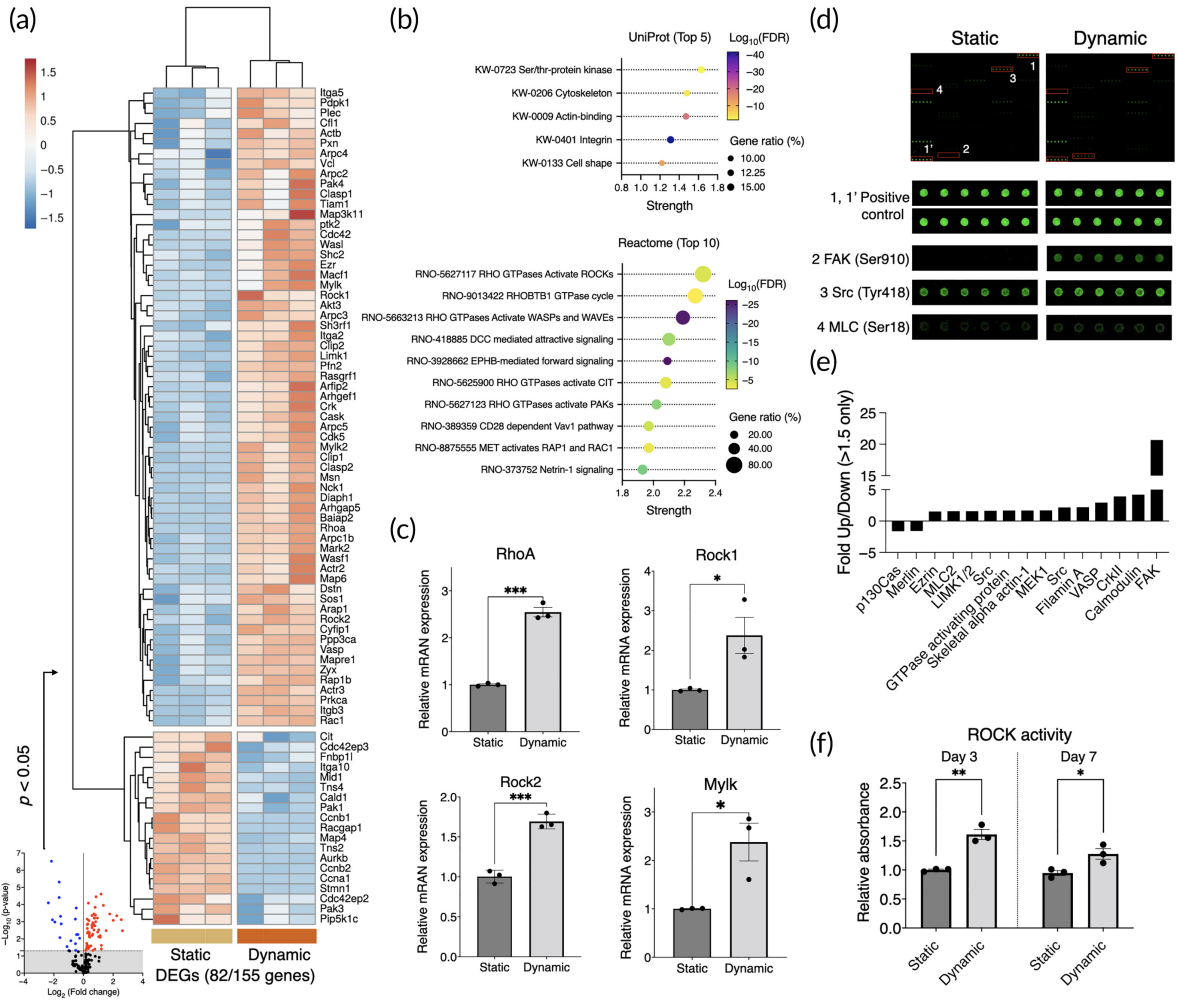
Cytoskeletal rearrangement and actomyosin contractility of BMSC in the dynamic condition. (a) Gene expression array of 155 markers encoding adhesion, migration, and cytoskeletal regulation factors. Differentially expressed genes (*p* < 0.05, DEGs) were plotted in the heatmap. Unit variance scaling was applied to ΔΔCT values, and rows were centered. (b) Gene set enrichment analysis using UniProt and Reactome database. Top 5 enriched keywords and top 10 enriched pathways were displayed. (c) RT‐qPCR analysis of Rho‐ROCK signaling down to myosin light chain kinase. (d, e) Antibody microarray of cytoskeletal regulators. (f) Enzymic activity of ROCK measured by phosphorylation of myosin phosphatase target subunit 1 (MYPT1). Statistical comparison between the static and dynamic conditions was performed by Student's *t*‐test. *p < 0.05; **p < 0.01; ***p < 0.001.

To evaluate the effect of perfusion culture on the cytoskeletal regulation, mRNA and protein expressions and enzymic activity governing cell adhesion, motility, and contractility were evaluated. Under the dynamic cell culture condition, BMSC differentially expressed genes encoding adhesion receptors (e.g., Itga2, Itga5, Itga10, and Itgb3), focal adhesion complex and actin binding proteins (e.g., Pkt2, Zyx, Pxn, Vcl, Tns2, Tns4, Vasp, and Zyx), and factors involved in Rho GTPase regulation and downstream signaling (RhoA, Rock1, Rock2, Rasgrf1, Racgap1, Rac1, Limk1, Cdc42) (Figure 3a). The gene set enrichment analysis using UniProt database identified that Ser/Thr protein kinase, cytoskeleton, actin‐binding, integrin, and cell shape were the highly enriched keywords. Reactome pathway analysis indicated that Rho GTPases and downstream signaling were highly enriched (Figure 3b). Validation by RT‐qPCR supported the activation of Rho‐ROCK signaling in the dynamic culture where RhoA (*p* = 0.00012), Rock1 (*p* = 0.039), Rock2 (*p* = 0.00061), and Mylk (*p* = 0.024) were significantly upregulated in the dynamic culture (Figure 3c). Cytoskeleton antibody microarray showed 20.7‐fold increase in the expression of focal adhesion in the dynamic culture accompanied by the minor upregulation of steroid receptor coactivator (Src: 1.64‐fold increase) and myosin regulatory light chain‐2 (MLC2: 1.57‐fold increase) (Figure 3d,e). The enzymic activity of ROCK significantly increased after 3 days (*p* = 0.002) and 7 days (*p* = 0.031) of perfusion culture, supporting the findings from the arrays (Figure 3f).

**FIGURE 4:**
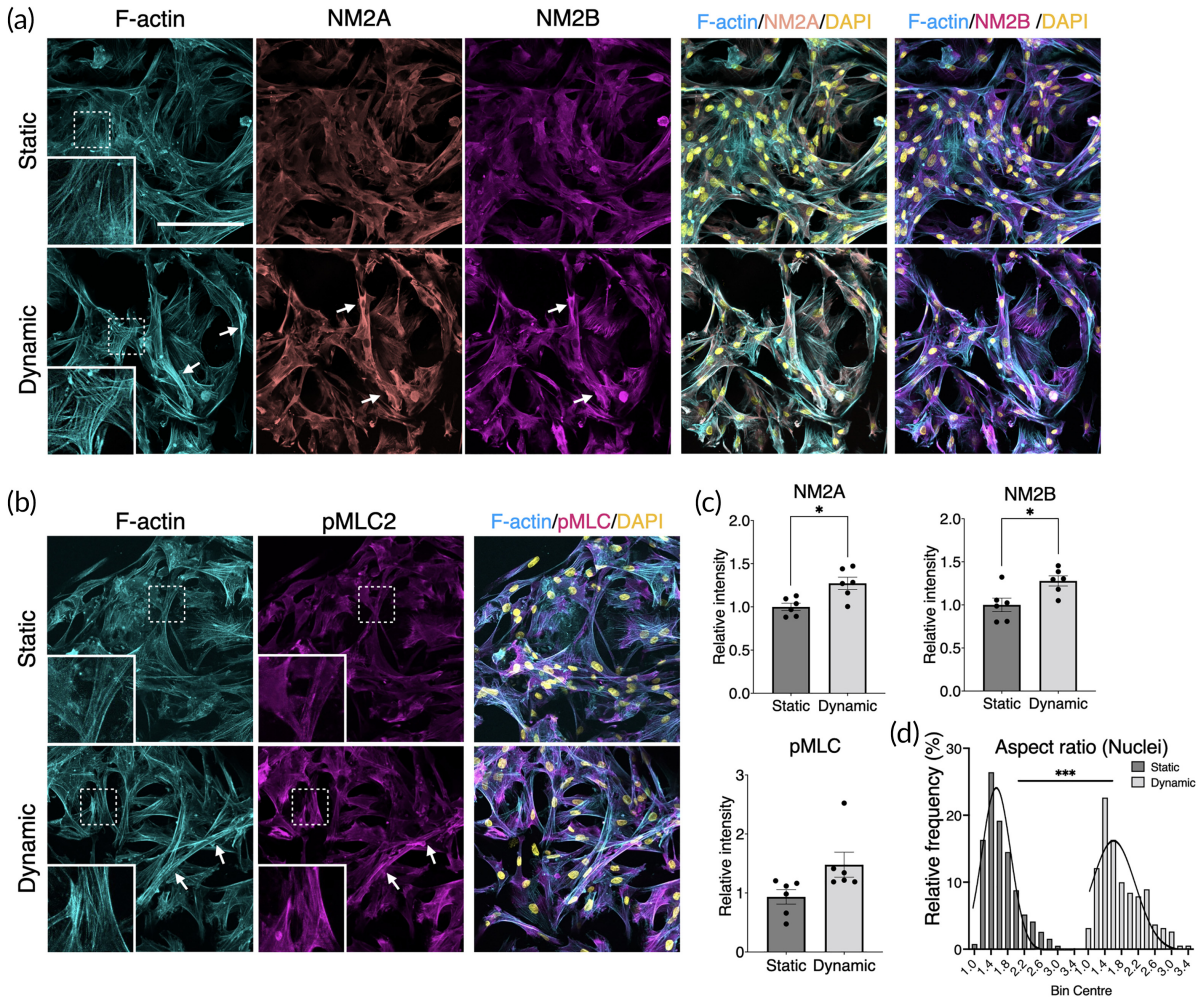
Immunofluorescence of actomyosin under static and dynamic cell culture. (a, b) In the dynamic culture condition, BMSC showed noticeable actomyosin contraction highlighted by highly polymerized filamentous actin (F‐actin), nonmuscle myosin 2A (NM2A), nonmuscle myosin 2B (NM2B), and phosphorylated myosin light chain 2 (pMLC2). Arrows indicate highly contracted sites. (c) Image quantification of NM2A, NM2B, and pMLC, showing enhanced expression. (d) Cell morphometric assessment by nuclear aspect ratio supported the evidence of cell contraction (*p* = 0.0006). The fitted distribution lines were generated by Gaussian distribution equation. Statistical comparison between the static and dynamic conditions was performed by Student's *t*‐test. *p < 0.05; ***p < 0.001.

Immunofluorescence confirmed the dynamic cytoskeletal rearrangement and contractility under perfusion culture (Figure 4a–d). BMSC under the dynamic condition showed enhanced actin polymerization, nonmuscle myosin (NM2A/2B), and phosphorylation of myosin light chain2 (MLC2), resulting in more elongated morphology compared to the static counterpart (*p* = 0.0006).

### 3.3 Cell contractility is necessary for maintaining cell growth under perfusion

**FIGURE 5:**
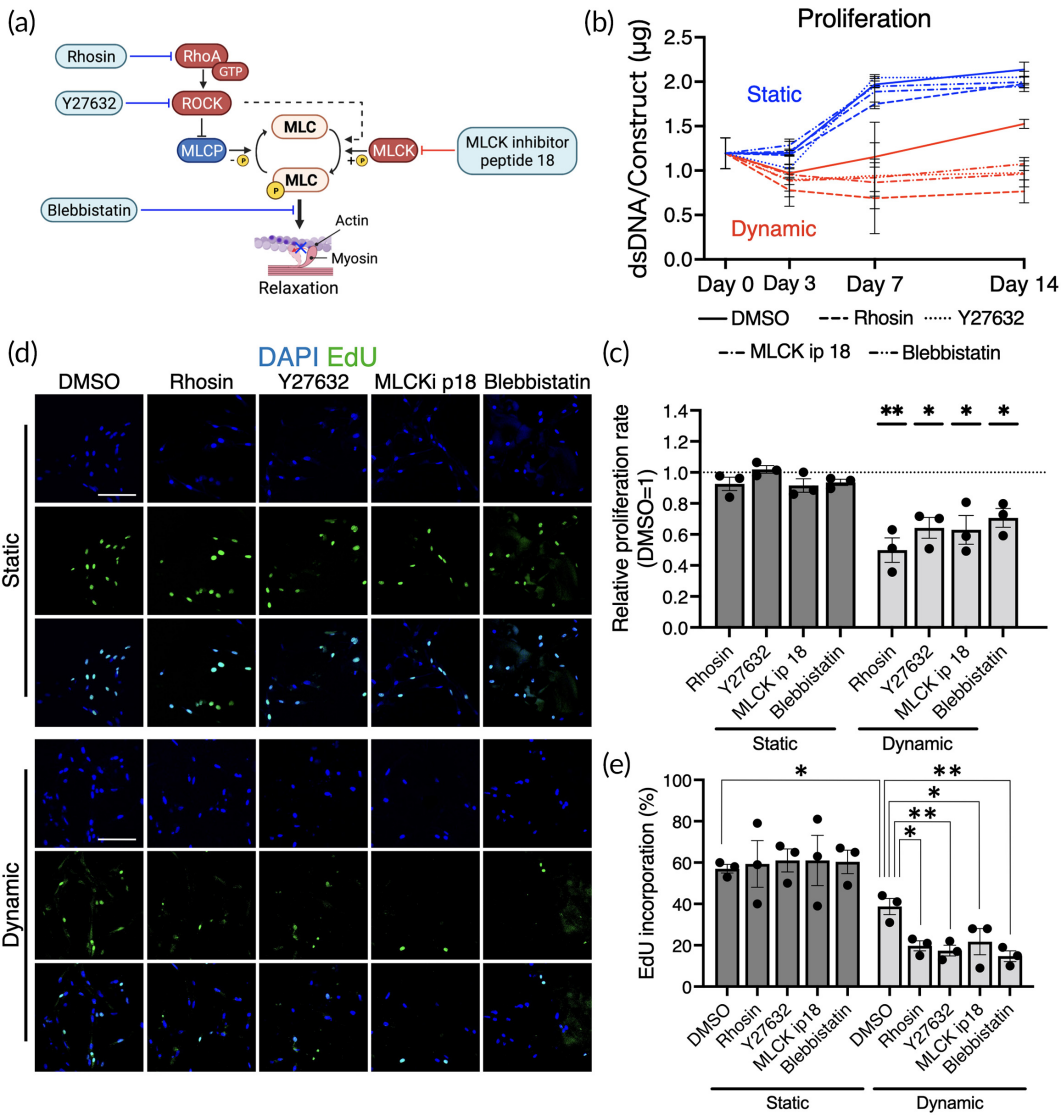
Cell growth under perfusion with/without inhibitors of actomyosin contractility. (a) Rhosin chloride (Rhosin), Y27632 dihydrochloride (Y27632), MLCK inhibitory peptide 18 (MLCK ip 18), and Blebbistatin (203390) were added to the culture medium during the dynamic cell culture, leading forcible cell relaxation. (b, c) The quantification of double strand DNA (dsDNA) demonstrated the cells in the dynamic culture were less proliferative compared to the static counterpart. The inhibition of actomyosin contractility mitigated cell proliferation in the dynamic but not static conditions. Dunnett's multiple comparison was performed by comparing to DMSO control within static and dynamic conditions. (d, e) EdU (5‐ethynyl‐2′‐deoxyuridine) incorporation during 24 hours of cell culture, detected by confocal microscope, decreased in the dynamic condition, particularly in the presence of the inhibitors. Statistical comparison between the static and dynamic conditions without the inhibitors was performed by Student's *t*‐test and within the groups by ANOVA followed by Dunnett's multiple comparison. *p < 0.05; **p < 0.01. Scale bar = 100 μm.

To evaluate the role of cell contractility in cell growth during dynamic cell culture, four different small molecule inhibitors (i.e., Rhosin, Y27632, MLCK ip 18, and Blebbistatin) were used to drive cell relaxation (Figure 5a). In the present system, we found that perfusion culture slowed cell growth (Figure 5b,c). At concentrations that did not affect dsDNA yield in the static condition, the inhibitors did prevent cells from proliferating in the dynamic cell culture (Day 3: *p* = 0.0013, Days 7–14: *p* < 0.0001). EdU incorporation assays confirmed that the reduced dsDNA yield was due to cell cycle arrest (Figure 5d,e). With or without inhibitors, in the static condition, approximately 60% of the cells incorporated EdU in 24 hours, but only 40% did so in the dynamic condition (*p* = 0.015). Proliferation was further diminished by the inhibitors, with < 20% of the cells found to be proliferative (DMSO VS Rhosin: *p* = 0.019; Y27632: *p* = 0.0098; MLCK ip 18: *p* = 0.035; Blebbistatin: *p* = 0.0046). When actomyosin contraction was pharmacologically induced in a static culture by dosing Narciclasine or Calyculin A for 7 days, cell proliferation also significantly decreased (Figure S3A). Together, these results suggest that excess cellular contractility slows proliferation in static culture but is necessary for maintaining their proliferative status under 3D perfusion culture.

### 3.4 Mechanically induced osteogenic gene expression profile differed from chemically induced osteogenesis

**FIGURE 6:**
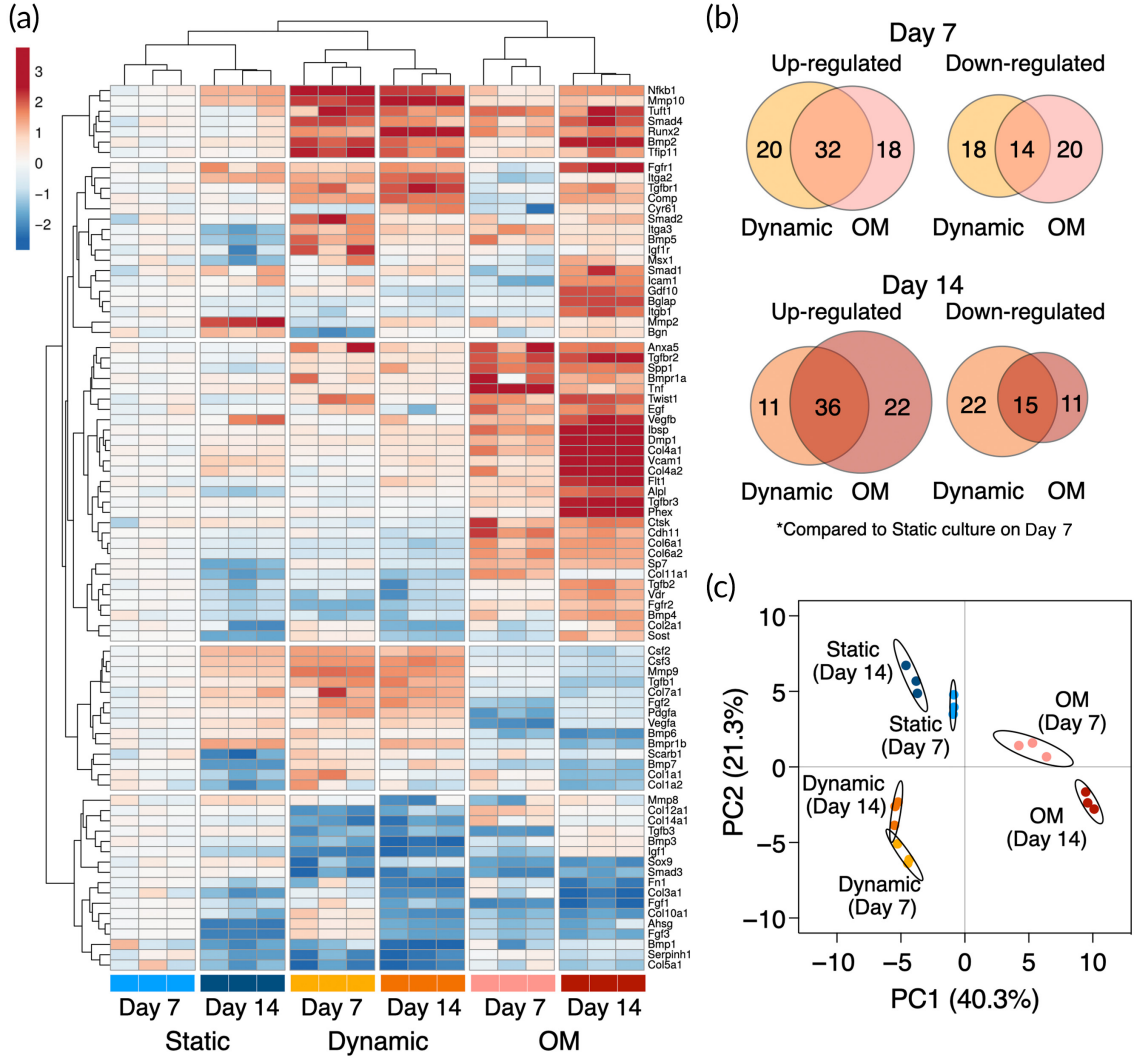
Osteogenic gene expression profile of mechanically stimulated BMSC. (a) Heatmap of mRNA expression levels showing the expression pattern of 84 osteogenesis‐associated markers in the static, dynamic, and chemically stimulated conditions by osteoinductive medium (OM). Both rows and columns are clustered using Euclidean distance and complete linkage. Unit variance scaling was applied to log_2_FC values. (b) Venn diagrams presenting number of differentially expressed gene on Days 7 and 14. (c) Principal component analysis (PCA) of osteogenic gene expression pattern. X and Y axis show principal component 1 and principal component 2 that explain 40.3% and 21.3% of the total variance, respectively. Prediction ellipses are such that with probability 0.95, a new observation from the same group will fall inside the ellipse. Samples are colored according to their conditions and timepoints.

To elucidate the osteogenic profile of the mechanically stimulated BMSC, the expression of 84 genes associated with osteogenesis was compared between the dynamic condition, the static condition as a negative control, and the chemically osteoinductive condition (i.e., OM) as a positive control. In the dynamic condition, the BMSC upregulated 52 and 47 osteogenesis‐related markers on Days 7 and 14, respectively, compared to the static counterpart (Figure 6a,b). Active metabolism of extracellular matrices during the dynamic culture featured the expression profile on Day 7 where the upregulation of mRNA encoding the major components of bone extracellular matrices such as collagen (Col1a1, Col1a2, Col2a1, Col4a1, Col7a1, and Col10a1) was observed simultaneously with the robust upregulation of matrix metalloproteinases (Mmp9, 61.62‐fold, *p* = 0.00010; Mmp10, 149.66‐fold, *p* = 0.0069). This trend was not observed in the OM group. The upregulated genes in the dynamic condition on Day 14 included a key transcriptional factor for osteogenesis, Runx2 (2.71‐fold, *p* < 0.0001), as well as common late differentiation markers such as dentin matrix acidic phosphoprotein 1 (Dmp1, 4.38‐fold, *p* = 0.0012), alkaline phosphatase (Alp, 1.21‐fold, *p* = 0.038), osteopontin (Spp1, 3.28‐fold, *p* = 0.0025), and bone sialoprotein (Ibsp, 5.15‐fold, *p* = 0.0021) compared to the static counterpart. The expression of Runx2 was significantly higher in the dynamic condition than the OM group on Day 7 (1.65‐fold, p‐0.00077) and Day 14 (1.54‐fold, *p* = 0.00033). Additionally, fibroblast growth factors (Fgfs: Fgf1, Fgf2, and Fgf3), transforming growth factor‐β superfamily (Tgfs: Tgfb1, Tgfb2; Bmps: Bmp2, Bmp5, Bmp6, and Bmp7) and its transducer (Smad2, Smad4), and colony‐stimulating factors (Csfs: Csf2, Csf3) were found upregulated in the dynamic condition over the period. Noteworthily, a late osteogenic marker, osteocalcin (Bglap), was downregulated in both dynamic and OM groups on Day 7 compared to the static counterpart (dynamic, 0.14‐fold, *p* = 0.0057; OM, 0.58‐fold, *p* = 0.090). It remained downregulated in the dynamic condition (0.26‐fold, *p* = 0.091) but was significantly upregulated in the OM group (305‐fold, *p* < 0.0001) on Day 14.

Despite some quantitative differences, the overall osteogenic profile in the dynamic condition remained comparable between Days 7 and 14, whereas an increase in a number of upregulated genes (50 genes on Day 7 and 58 genes on Day 14) as well as a significant increase in the quantity of the expression was observed in the OM group. This would indicate that, although perfusion culture stimulates the expression of some osteogenesis‐related genes, prolonged perfusion culture may not maturate osteogenicity, at least within the 14‐days period. Principal components analysis (PCA) highlighted the unique osteogenic profile of the mechanically stimulated cells with a clear separation from the static and chemically induced groups (Figure 6c). The clusters of perfusion‐induced osteogenesis were indeed shifted orthogonally to conventional osteogenic differentiation, but the subsequent experiments revealed the expression patterns still supported conventional osteogenic functionality.

### 3.5 Mechanically induced osteoblastic functionality is governed by Rho‐ROCK signaling and cell contractility

To investigate whether (1) the mechanically induced osteogenic profile was governed by cell contraction under perfusion and (2) the mechanically stimulated BMSC practically possessed the osteoblastic phenotype, osteogenic differentiation was further evaluated with and without the cell contraction inhibitors.

**FIGURE 7:**
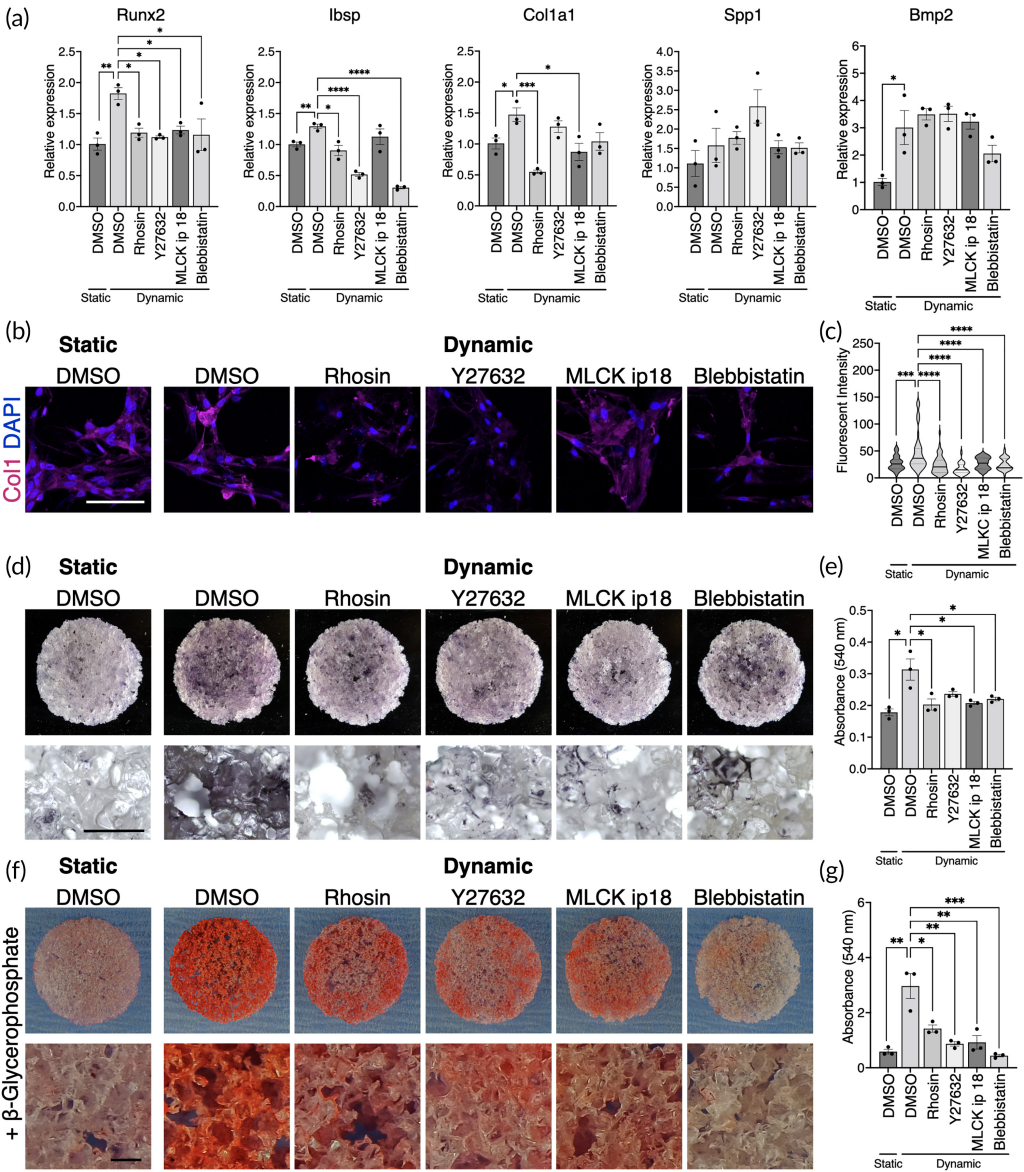
Mechanically induced osteogenesis is dependent on actomyosin contractility (a) mRNA expression levels of osteogenic markers in the static and dynamic conditions with/without the inhibitors of actomyosin contractility. (b) Immunofluorescence of type 1 collagen and (c) quantification of fluorescent intensity. (d) Alkaline phosphatase staining and (e) quantification of extracted substrate. (f) Alizarin red S staining and (g) quantification of extracted dye. The samples for the mineralization assay were cultured in the presence of β‐glycerophosphate as a source of phosphate ions. Statistical comparison between the static and dynamic condition was performed by the Student's *t*‐test and comparison among pharmacologically treated groups were by ANOVA followed by Dunnett's multiple comparison. *p < 0.05; **p < 0.01; ***p < 0.001; ****p < 0.0001. White scale bar = 100 μm, black scale bar = 500 μm.

As the osteogenic profiling revealed, the expression of Runx2 (1.82‐fold increase, *p* = 0.0038), Ibsp (1.29‐fold increase, *p* = 0.0053), Cal1a1 (1.47‐fold increase, *p* = 0.030), Spp1 (1.57‐fold increase, *p* = 0.45), and Bmp2 (3.0‐fold increase, *p* = 0.035) increased in the dynamic condition compared to the static counterpart on Day 7 (Figure 7a). The use of the inhibitors fully or partially counteracted the upregulation of Runx2, Ibsp, and Col1a1 but not Spp1. The mitigation of mechanically induced Bmp2 expression by the inhibitors was not statistically significant.

For the functionality assessment of the stimulated BMSC, collagen matrix formation, alkaline phosphatase activity, and calcium deposition were evaluated. While the cells in the static condition secreted type 1 collagen faintly, clusters of the cells significantly enriched collagen production in the dynamic condition (Figure 7b,c). This was depicted as an outstanding peak in the dynamic condition by the quantification (*p* = 0.0002). The inhibitors, particularly Y27632, disrupted the perfusion‐enhanced collagen secretion. Similarly, ALP staining showed the upregulated ALP activity in the dynamic condition compared to the static counterpart (*p* = 0.0019), which was, however, negated by the inhibitors (Figure 7d,e). Calcium deposition was evaluated as an indicator of maturation of osteogenic differentiation by adding beta‐glycerophosphate as a source of phosphate ions in the growth medium. Remarkably, the mechanically stimulated cells successfully deposited calcium nodes on the scaffolds while beta‐glycerophosphate alone was not sufficient to induce mineralization in the static condition (*p* = 0.0066) (Figure 7f,g). Mineralization was attenuated by the inhibitors, particularly blebbistatin, completely masking the pro‐osteogenic effect of dynamic cell culture. Taken together, dynamic cell culture in the perfusion bioreactor was sufficient to induce BMSC osteogenesis, which was dependent on enhanced cellular contractility.

## 4 DISCUSSION

Using a bioreactor for 3D cell culture is a promising strategy to improve the efficiency of cell therapy in bone tissue engineering. Based on credible evidence identified in conventional monolayer cell culture studies that mechanical stimulation promotes BMSC osteogenesis, the concept has been brought to 3D systems for translational research. However, due to the complexity of 3D cell culture in a dynamic environment, the knowledge gained so far seems fragmented, particularly when in the absence of osteoinductive medium. This is because in different bioreactor systems developed for bone tissue engineering not only dynamic conditions but also basic cell culture factors such as volume of medium, humidification method, and static pressure may differ.^39^, ^43^, ^44^ The chemical and physical properties of scaffolding materials may also synergistically influence cellular behaviors in a dynamic environment. Additionally, the precise evaluation of fluid dynamics with 3D scaffolds requires high computational cost, which may not always be affordable.^45^ Consequently, flow is often described only by flow rate (e.g., mL/min) or pump speed (e.g., rpm), but not the magnitude of fluid force exerted on cells.^46^ These factors make it difficult to interpret experimental findings from different models. In the present study by culturing BMSC in the perfusion bioreactor, the comprehensive analyses of cell behaviors under perfusion culture were performed including detailed CFD analysis, cell morphological assessment, cell growth, and mechanically induced osteogenesis in the absence of osteoinductive medium.

In the present study, the optimal flow rate was preliminarily determined as 1.0 mL/min in the system. The flow rate corresponded to estimated shear stress of approximately 0.5–5 mPa (mean 1.56 mPa) to which most of BMSC on the scaffolds were exposed. Fluid paths were estimated to be distributed uniformity throughout the constructs, which is an important aspect of the dynamic culture to improve mass transfer from/to the constructs although a magnitude of fluid shear varied spatially. BMSC and osteoblastic cells are reportedly more vulnerable to fluidic stimulation on 3D porous scaffolds compared to the 2D monolayered counterpart, and excessive shear stress can lead to apoptosis.^21^, ^37^, ^47^, ^48^, ^49^, ^50^, ^51^, ^52^ This could be because cell morphological adaptation and migration are restricted to the geometry of scaffolds when fluid stimuli act in multiple directions.^53^ It was therefore no surprise that the shear stress used in our study, although considerably lower than that reported to promote osteogenesis in the conventional 2D systems, was sufficient to provoke dynamic cellular behaviors.

Under this level of perfusion, BMSC clearly showed a sign of morphological change with a number of upregulated factors for cell morphogenesis including surface mechanoreceptors, focal adhesion complex, and the direct inducers of actomyosin contractility. Elevated actomyosin contractility directly links to BMSC fate determination as a process and an endpoint. The angular, elongated morphology and increased rheological stiffness of BMSC is associated with high expression of actomyosin and focal adhesion and with osteoblast fate, whereas rounded morphology, low cell stiffness and lower actomyosin and focal adhesion expression is associated with adipogenic differentiation.^54^, ^55^, ^56^ Therefore, it would be reasonable to mention that the cytoskeletal features exhibited by the BMSC in the present system favored osteogenic differentiation.

Our results indicated that perfusion culture delayed cell proliferation, possibly mediated by cell contraction. The reported effect of perfusion culture on cell proliferation is inconsistent in the published literature in both 2D and 3D systems. There are studies reporting mitogenic effects,^11^, ^12^, ^30^, ^33^, ^57^, ^58^, ^59^, ^60^ anti‐mitogenic effects,^15^, ^16^, ^32^, ^36^, ^61^ and no effect,^14^, ^34^ while all promoting osteogenic phenotypes. The inconsistency could arise from differences in the magnitude of shear stress, the type of cells used, measurement methodology, and/or the efficiency of passive nutrient transport within scaffolding material since low‐diffusive scaffolds would benefit from perfusion more than highly permeable materials with the respect to nutrient transport. Previous studies in a 2D cell culture model demonstrated that laminar shear stress reduces MSC proliferation in a dose‐dependent manner by arresting cell cycle at G_0_/G_1_ phases.^51^, ^62^ In the present study, where the highly porous scaffolds with macropores were used, the reduction of cell proliferation could be attributed largely to shear stress on the cells. The cell contractility inhibitors of Rho, ROCK, MLCK, and myosin II blocked cell cycle further and hindered cell proliferation in the dynamic condition. Taken together, increased cell contraction caused by shear stress was associated with reduced cell proliferation, but contractility was indeed necessary for maintaining proliferation under flow.

Mechanically induced or promoted osteogenic differentiation is often measured by increased ALP activity and the upregulation of key osteogenic markers, such as Runx2. However, the term “osteogenic differentiation” has been used casually without sufficient definition, and the overall osteogenic profile under mechanical stimulation has not hitherto been adequately described. The present study highlighted the unique osteogenic profile of BMSC stimulated by fluid flow. Even in the absence of the osteoinductive supplements, fluid shear stress stimulated the mRNA expression of osteogenic markers, such as Runx2, Alp, and Ibsp. Interestingly, the expression of Runx2 was significantly higher in the dynamic condition than the OM group, and signaling molecules such as Bmps, Fgfs, Csfs, and Tgfs were differentially expressed. This has revealed that fluid shear stress may induce osteogenic differentiation differently from the conventionally described differentiation by the OM. Nevertheless, the functionality gained by the mechanically stimulated BMSC reconciled with typical preosteoblast‐like phenotypes, featured by an increase in type 1 collagen formation and ALP activity. It is generally considered that, in the absence of the osteoinductive supplements, mechanical stimulation promotes ALP activity and the expression of early osteogenic markers, but it is not sufficient to induce osteoblastic maturation characterized by the upregulation of Bglap and mineralization.^21^, ^23^, ^24^, ^57^, ^58^, ^59^ Here, by adding beta‐glycerophosphate as a source of inorganic phosphate ions in the growth medium, we demonstrated that the mechanically stimulated BMSC were capable of depositing calcium nodes despite a lack of Bglap expression. In fact, the initiation of mineralization precedes the significant upregulation of Bglap in vitro,^38^ suggesting that the mechanically stimulated BMSC indeed possessed the phenotype of moderately mature osteoblastic cells. The inhibition of actomyosin contractility under flow significantly mitigated their osteogenic functionality, confirming that cytoskeletal rearrangement via Rho‐ROCK signaling is necessary for the osteogenic responses triggered by fluidic stimuli in the absence of osteoinductive medium. Nevertheless, the mechanically upregulated osteogenic markers were not consistently suppressed by the inhibitors. Previous studies have shown that fluid shear stress at physiological levels modulates various pathways such as ERK/MAPK signaling, P38 signaling, TGFβ signaling, Hedgehog signaling, Notch signaling, and Wnt signaling pathways in MSC as well as osteoblasts and osteocytes.^16^, ^21^, ^63^, ^64^, ^65^ The regulation of osteogenic genes is multifactorial, and these pathway may interplay with osteogenic genes differently, which may bypass the Rho‐ROCK‐mediated cell contraction. Although this study does not enter the debate as to whether Rho‐ROCK activation and/or cell contraction are sufficient to induce osteogenesis, we observed that the pharmacological activation of Rho by Narciclasine significantly modulated the mRNA expression level of Runx2 and Bmp2 (Figure S3B). The upregulation of Bmp2 was also induced by the inhibition of myosin phosphatase, Calyculin A. However, both drugs significantly decreased Col1a1 expression, and we did not find a rational correlation between cell contractility and ALP activity from the preliminary data (Figure S3C,D). It would be therefore reasonable to conclude that osteogenic induction by fluid stimuli is governed by, but not limited to, Rho‐ROCK‐mediated cytoskeletal modulation.

Translation of knowledge in mechanobiology into the field of tissue engineering has successfully paved the way for the development of prominent dynamic cell culture platforms for regenerative medicine. This will facilitate effective 3D cell culture and improve cell‐based therapy for bone regeneration. To bring mechanically stimulated cells into clinical settings, it is necessary to accurately grasp cell response to dynamic environment for safety and efficacy. Each system is unique, and bioreactor designs, material selection, scaffold geometry, supplemented drugs, cell origin, and other environmental factors synergistically or reciprocally interact with resulting cell behaviors.^66^, ^67^ More to the point, biological variables attributable to donors such as age, gender, systemic conditions, and epigenetics need to be taken into consideration when it comes to clinical application.^68^ In the study, male Lewis rats were used as a source of BMSC because of the abundancy of BMSC compared to female animals.^69^ Admittedly, our observation needs to be verified by human cells isolated from donors with various background. Therefore, further in‐depth studies using diverse experimental settings are required to fully elucidate mechanically induced osteogenesis and its potential for future clinical translation.

## AUTHOR CONTRIBUTIONS

**Shuntaro Yamada:** Conceptualization (equal); data curation (equal); formal analysis (equal); funding acquisition (supporting); investigation (lead); methodology (lead); resources (equal); software (equal); validation (equal); visualization (equal); writing – original draft (lead); writing – review and editing (equal). **Mohammed Yassin:** Conceptualization (equal); formal analysis (equal); investigation (equal); methodology (equal); supervision (equal); writing – original draft (equal); writing – review and editing (equal). **Francesco Torelli:** Investigation (supporting); methodology (supporting); writing – original draft (supporting); writing – review and editing (equal). **Jan Hansmann:** Conceptualization (equal); formal analysis (equal); investigation (equal); methodology (equal); project administration (equal); resources (equal); software (equal); supervision (equal); validation (equal); writing – original draft (supporting); writing – review and editing (equal). **Jeremy Green:** Conceptualization (equal); formal analysis (equal); methodology (equal); writing – original draft (equal); writing – review and editing (equal). **Thomas Schwarz:** Methodology (equal); resources (equal); writing – original draft (equal); writing – review and editing (equal). **Kamal Mustafa:** Conceptualization (equal); formal analysis (equal); funding acquisition (lead); investigation (equal); methodology (equal); project administration (lead); resources (equal); supervision (lead); validation (equal); writing – original draft (equal); writing – review and editing (equal).

## CONFLICT OF INTEREST STATEMENT

The authors declare no potential conflicts of interest with respect to the authorship and/or publication of this article.

### PEER REVIEW

The peer review history for this article is available at https://www.webofscience.com/api/gateway/wos/peer‐review/10.1002/btm2.10509.

## Supporting information


**Fig. S1:** Optimization of inhibitors for suppressing actomyosin contractility (A) Rhosin chloride (Rhosin), Y27632 dihydrochloride (Y27632), MLCK inhibitory peptide 18 (MLCK ip 18), and Blebbistatin (203390) were applied to inhibit Rho, ROCK, myosin light chain kinase (MLCK), and myosin II, respectively, to forcibly trigger cell relaxation. (B–J) Cell growth, viability, and actomyosin contraction were evaluated to optimize the working concentrations. *p < 0.05; **p < 0.01; ***p < 0.001; ****p < 0.0001. Scale bar = 100 μm.

**Fig. S2:** Optimization of enhancers of actomyosin contractility (A) Narciclasine and Calyculin A were applied to activate Rho and to inhibit myosin light chain phosphatase (MLCP) to forcibly trigger cell contraction. (B–F) Cell growth, viability, and actomyosin contraction were evaluated to optimize the working concentrations. *p < 0.05; **p < 0.01; ***p < 0.001; ****p < 0.0001. Scale bar = 100 μm.

**Fig. S3:** BMSC Growth and osteogenic properties under pharmacologically triggered actomyosin contraction BMSC were treated with 0.05 nM and 0.2 nM Calyculin A (CalA) and 1 nM and 5 nM Narciclasine (Narc) (A) Quantification of double‐strand DNA (dsDNA) after 7 days of static culture. (B) mRNA expression of putative osteogenic markers after 7 days of static culture. (C, D) Alkaline phosphatase staining and quantification of extracted substrate after 14 days of static culture. *p < 0.05; **p < 0.01; ***p < 0.001; ****p < 0.0001.

**Table S1:** A set of genes and primers used for the assessment of cytoskeletal rearrangement under perfusion

**Table S2:** A set of genes and primers used for the assessment of osteogenic profile under perfusion

**Table S3:** A set of genes and primers used as an endogeneous control

## Data Availability

The raw data supporting the conclusion of this article will be made available by the authors, without undue reservation.
