# Supplementary material for: Unique osteogenic profile of bone marrow stem cells stimulated in perfusion bioreactor is Rho‐ROCK‐mediated contractility dependent
Source: Bioeng Transl Med. 2023 Mar 17;8(3):e10509. doi: 10.1002/btm2.10509 (PMC10189446; doi:10.1002/btm2.10509)
Supplement: Supplementary file 1 — Fig. S1: Optimization of inhibitors for suppressing actomyosin contractility (A) Rhosin chloride (Rhosin), Y27632 dihydrochloride (Y27632), MLCK inhibitory peptide 18 (MLCK ip 18), and Blebbistatin (203390) were applied to inhibit Rho, ROCK, myosin light chain kinase (MLCK), and myosin II, respectively, to forcibly trigger cell relaxation. (B–J) Cell growth, viability, and actomyosin contraction were evaluated to optimize the working concentrations. *p < 0.05; **p < 0.01; ***p < 0.001; ****p < 0.0001. Scale bar = 100 μm. [file BTM2-8-e10509-s003.pdf]

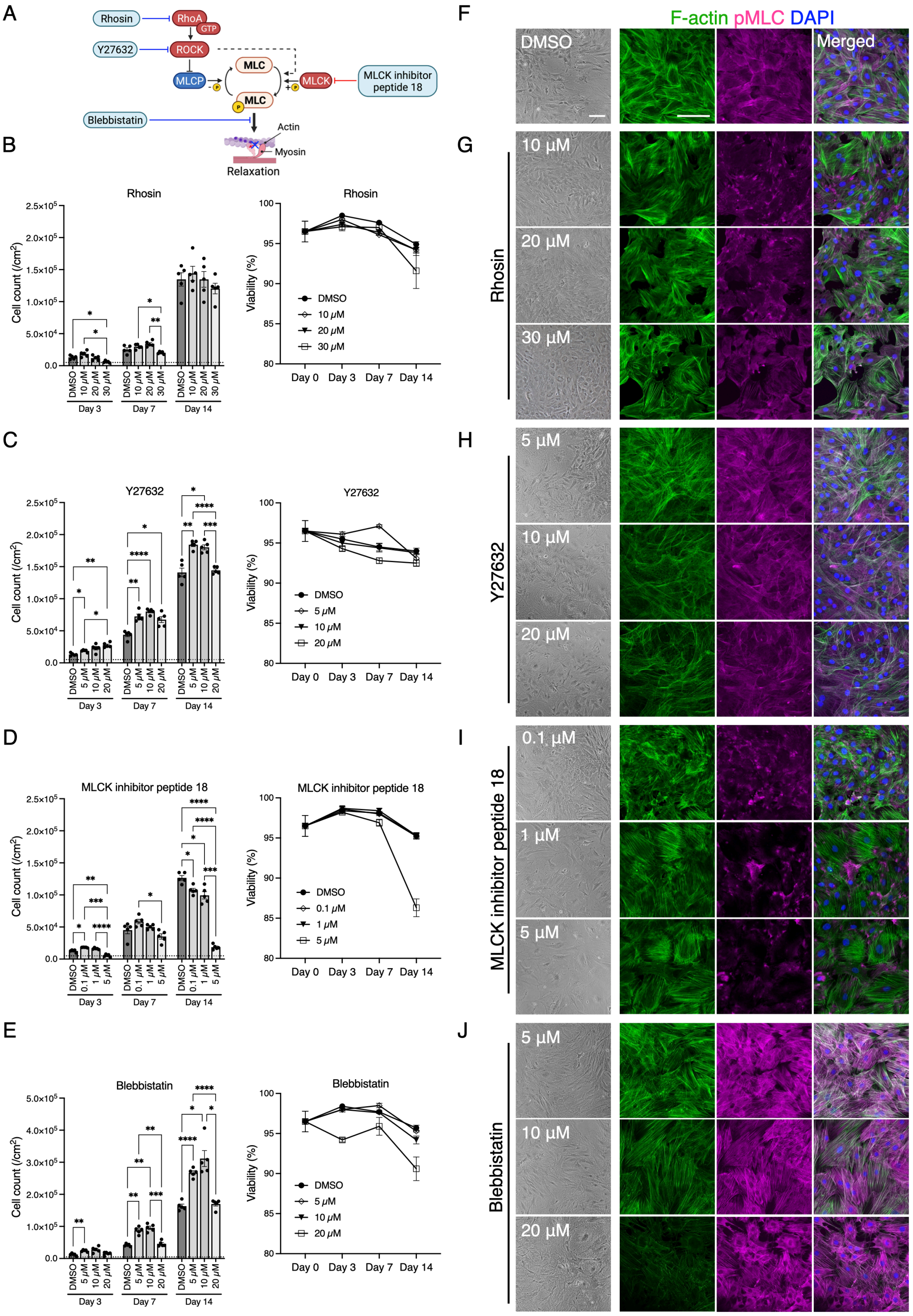

Fig. S1 Optimization of inhibitors for suppressing actomyosin contractility

(A) Rhosin chloride (Rhosin), Y27632 dihydrochloride (Y27632), MLCK inhibitory peptide 18 (MLCK ip 18), and Blebbistatin (203390) were applied to inhibit Rho, ROCK, myosin light chain kinase (MLCK), and myosin II, respectively to forcibly trigger cell relaxation. (B-J) Cell growth, viability, and actomyosin contraction were evaluated to optimize the working concentrations. * p<0.05, ** p<0.01, *** p<0.001, **** p<0.0001. Scale bar = 100 μm.
