# Supplementary material for: Unique osteogenic profile of bone marrow stem cells stimulated in perfusion bioreactor is Rho‐ROCK‐mediated contractility dependent
Source: Bioeng Transl Med. 2023 Mar 17;8(3):e10509. doi: 10.1002/btm2.10509 (PMC10189446; doi:10.1002/btm2.10509)
Supplement: Supplementary file 2 — Fig. S2: Optimization of enhancers of actomyosin contractility (A) Narciclasine and Calyculin A were applied to activate Rho and to inhibit myosin light chain phosphatase (MLCP) to forcibly trigger cell contraction. (B–F) Cell growth, viability, and actomyosin contraction were evaluated to optimize the working concentrations. *p < 0.05; **p < 0.01; ***p < 0.001; ****p < 0.0001. Scale bar = 100 μm. [file BTM2-8-e10509-s002.pdf]

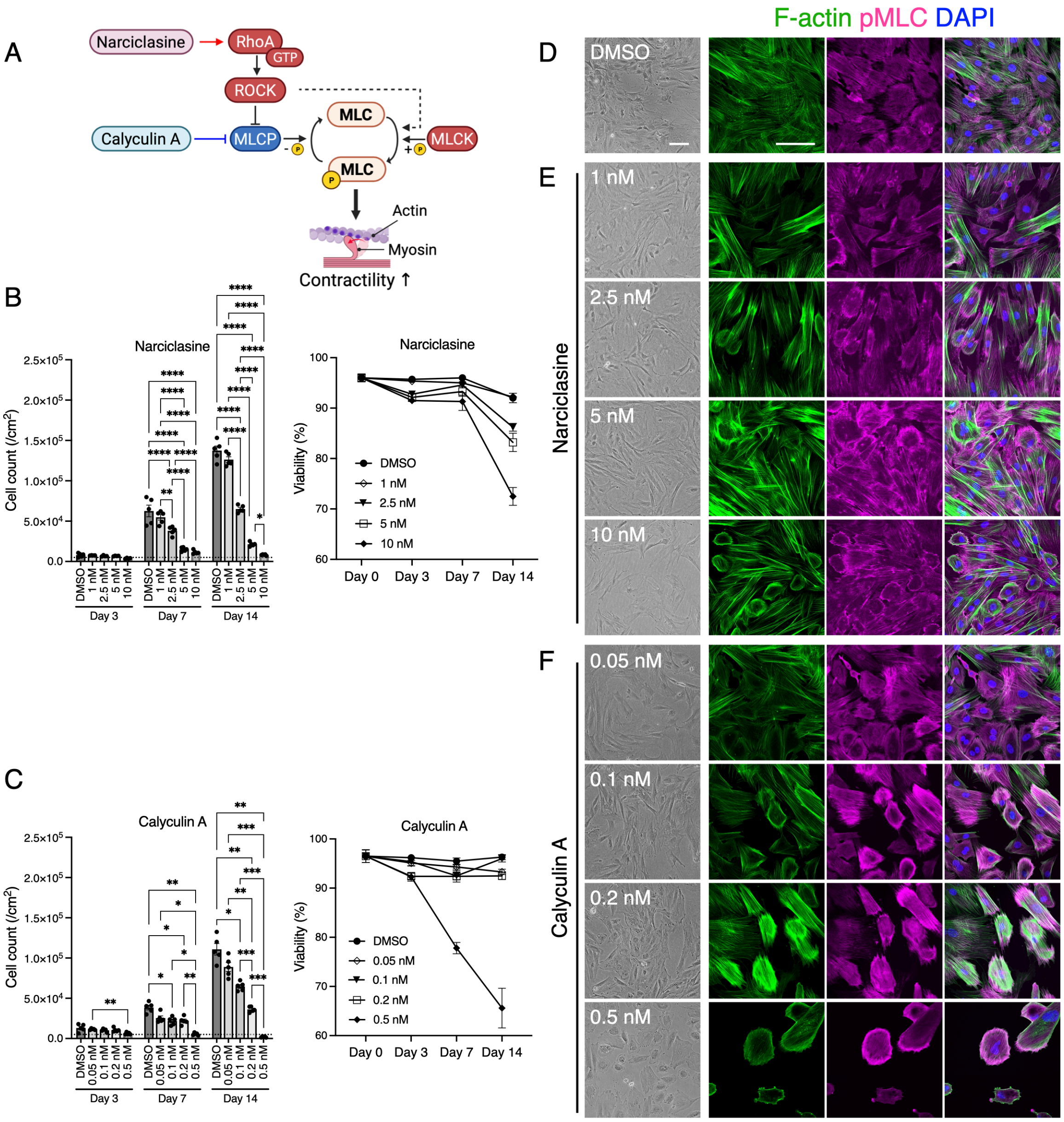

Fig. S2 Optimization of enhancers of actomyosin contractility

(A) Narciclasine and Calyculin A were applied to activate Rho and to inhibit myosin light chain phosphatase (MLCP) to forcibly trigger cell contraction. (B-F) Cell growth, viability, and actomyosin contraction were evaluated to optimize the working concentrations. * $p<0.05$, ** $p<0.01$, *** $p<0.001$, **** $p<0.0001$. Scale bar = 100 μm.
