# Supplementary material for: Unique osteogenic profile of bone marrow stem cells stimulated in perfusion bioreactor is Rho‐ROCK‐mediated contractility dependent
Source: Bioeng Transl Med. 2023 Mar 17;8(3):e10509. doi: 10.1002/btm2.10509 (PMC10189446; doi:10.1002/btm2.10509)
Supplement: Supplementary file 3 — Fig. S3: BMSC Growth and osteogenic properties under pharmacologically triggered actomyosin contraction BMSC were treated with 0.05 nM and 0.2 nM Calyculin A (CalA) and 1 nM and 5 nM Narciclasine (Narc) (A) Quantification of double‐strand DNA (dsDNA) after 7 days of static culture. (B) mRNA expression of putative osteogenic markers after 7 days of static culture. (C, D) Alkaline phosphatase staining and quantification of extracted substrate after 14 days of static culture. *p < 0.05; **p < 0.01; ***p < 0.001; ****p < 0.0001. [file BTM2-8-e10509-s001.pdf]

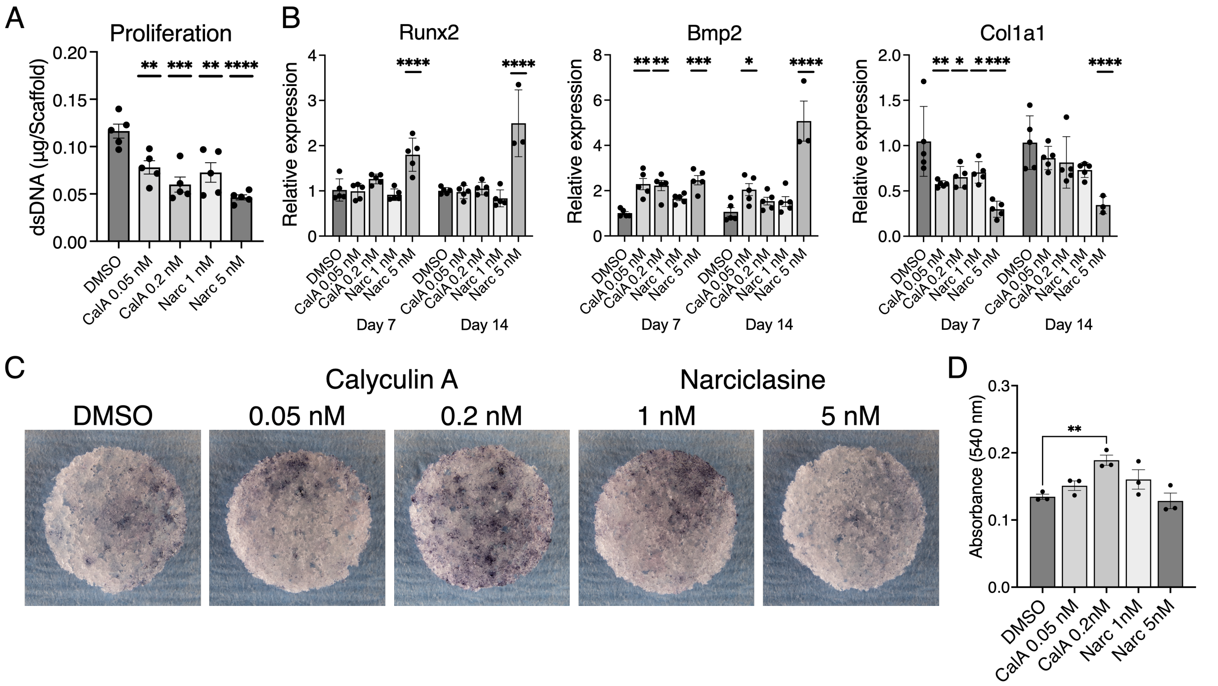

Fig. S3 BMSC Growth and osteogenic properties under pharmacologically triggered actomyosin contraction

BMSC were treated with 0.05 nM and 0.2 nM Calyculin A (CalA) and 1 nM and 5 nM Narciclasine (Narc) (A) Quantification of double-strand DNA (dsDNA) after 7 days of static culture. (B) mRNA expression of putative osteogenic markers after 7 days of static culture. (C, D) Alkaline phosphatase staining and quantification of extracted substrate after 14 days of static culture. * $p<0.05$, ** $p<0.01$, *** $p<0.001$, **** $p<0.0001$.
