# Supplementary material for: Unique osteogenic profile of bone marrow stem cells stimulated in perfusion bioreactor is Rho‐ROCK‐mediated contractility dependent
Source: Bioeng Transl Med. 2023 Mar 17;8(3):e10509. doi: 10.1002/btm2.10509 (PMC10189446; doi:10.1002/btm2.10509)
Supplement: Supplementary file 4 — Table S1: A set of genes and primers used for the assessment of cytoskeletal rearrangement under perfusion [file BTM2-8-e10509-s005.pdf]

Table. S1 A set of genes and primers used for the assessment of cytoskeletal rearrangement under perfusion

	Gene Symbol	Gene Name	TaqMan Assay ID	Amplicon Length (bp)
Target genes	Actn1	actinin, alpha 1	Rn00667357_m1	66
	Actn3	actinin alpha 3	Rn00591594_m1	88
	Actn4	actinin alpha 4	Rn00581554_m1	69
	Actr2	ARP2 actin-related protein 2 homolog	Rn01434079_m1	83
	Actr3	ARP3 actin-related protein 3 homolog	Rn01753774_m1	99
	Akt1	v-akt murine thymoma viral oncogene homolog 1	Rn00583646_m1	87
	Akt2	v-akt murine thymoma viral oncogene homolog 2	Rn00690900_m1	138
	Akt3	v-akt murine thymoma viral oncogene homolog 3	Rn00442194_m1	71
	Arap1	ArfGAP with RhoGAP domain, ankyrin repeat and PH domain 1	Rn01506749_m1	66
	Arfip2	ADP-ribosylation factor interacting protein 2	Rn01508475_m1	90
	Arhgap5	Rho GTPase activating protein 5	Rn03036858_s1	106
	Arhgap5	Rho GTPase activating protein 5	Rn03036858_s1	106
	Arhgdib	Rho, GDP dissociation inhibitor (GDI) beta	Rn01459333_m1	62
	Arhgef1	Rho guanine nucleotide exchange factor (GEF) 1	Rn00572505_m1	84
	Arhgef11	Rho guanine nucleotide exchange factor (GEF) 11	Rn00576857_m1	67
	Arpc1b	actin related protein 2/3 complex, subunit 1B	Rn00820959_g1	80
	Arpc2	actin related protein 2/3 complex, subunit 2	Rn01400361_m1	62
	Arpc3	actin related protein 2/3 complex, subunit 3	Rn01481651_m1	72
	Arpc4	actin related protein 2/3 complex, subunit 4	Rn01426286_m1	64
	Arpc5	actin related protein 2/3 complex, subunit 5	Rn01759260_m1	93
	Aurka	aurora kinase A	Rn00821847_g1	91
	Aurkb	aurora kinase B	Rn01460656_m1	95
	Aurkc	aurora kinase C	Rn01523742_g1	68
	Baiap2	BAI1-associated protein 2	Rn00589411_m1	97
	Bcar1	breast cancer anti-estrogen resistance 1	Rn00564003_m1	59
	Braf	B-Raf proto-oncogene, serine/threonine kinase	Rn01500563_m1	60
	Cald1	caldesmon 1	Rn00565719_m1	98
	Calm1	calmodulin 1	Rn00821407_g1	82
	Capn2	calpain 2	Rn00567422_m1	83
	Cask	calcium/calmodulin-dependent serine protein kinase	Rn00573365_m1	89
	Cav1	caveolin 1, caveolae protein	Rn00755834_m1	64
	Cav2	caveolin 2	Rn00590969_m1	72
	Cav3	caveolin 3	Rn00755343_m1	64
	Ccna1	cyclin A1	Rn01761351_m1	59
	Ccnb1	cyclin B1	Rn01494177_m1	97
	Ccnb2	cyclin B2	Rn02346769_g1	94
	Cdc42	cell division cycle 42	Rn00696671_m1	75
	Cdc42bpa	CDC42 binding protein kinase alpha	Rn00586520_m1	76
	Cdc42ep2	CDC42 effector protein (Rho GTPase binding) 2	Rn02376973_s1	63
	Cdc42ep3	CDC42 effector protein (Rho GTPase binding) 3	Rn01468719_m1	71
	Cdk5	cyclin-dependent kinase 5	Rn04219635_m1	95
	Cdk5r1	cyclin-dependent kinase 5, regulatory subunit 1 (p35)	Rn02132948_s1	134
	Cfl1	cofilin 1, non-muscle	Rn01501422_g1	77
	Cit	citron rho-interacting serine/threonine kinase	Rn00582449_m1	64
	Clasp1	cytoplasmic linker associated protein 1	Rn01408768_m1	59
	Clasp2	cytoplasmic linker associated protein 2	Rn00586859_m1	70
	Clip1	CAP-GLY domain containing linker protein 1	Rn00582048_m1	77
	Clip2	CAP-GLY domain containing linker protein 2	Rn00573248_m1	77
	Crk	v-crk avian sarcoma virus CT10 oncogene homolog	Rn00467066_m1	86
	Crkl	crk-like protein-like;v-crk avian sarcoma virus CT10 oncogene homolog-like	Rn01753203_m1	94
	Ctnnb1	catenin, beta 1	Rn00584431_g1	86
	Cttn	cortactin	Rn00573209_m1	67
	Cyfip1	cytoplasmic FMR1 interacting protein 1	Rn01409823_m1	59
	Cyfip2	cytoplasmic FMR1 interacting protein 2	Rn01471061_m1	65
	Diaph1	diaphanous-related formin 1	Rn01406955_m1	59
	Diaph1	diaphanous-related formin 1	Rn01406955_m1	59
	Dst	dystonin	Rn02110514_s1	93
	Dstn	destrin;destrin-like 1	Rn01415640_g1	89
	Ezr	ezrin	Rn01526350_m1	87
	Flna	filamin A, alpha	Rn01187530_m1	61
	Flnb	filamin B, beta	Rn01537993_m1	64
	Fnbp1l	formin binding protein 1-like	Rn01523678_m1	58
	Fscn2	fascin actin-bundling protein 2, retinal	Rn01470865_m1	61
	Fyn	FYN proto-oncogene, Src family tyrosine kinase	Rn00562616_m1	105
	Grb2	growth factor receptor bound protein 2	Rn01471333_g1	142
	Gsk3b	glycogen synthase kinase 3 beta	Rn00583429_m1	71
	Gsn	gelsolin	Rn01438922_m1	129
	Hras	Harvey rat sarcoma virus oncogene	Rn01479663_gH	72
	Ilk	integrin-linked kinase	Rn00591471_m1	70
	Iqgap1	IQ motif containing GTPase activating protein 1	Rn01478183_m1	62
	Itga1	integrin, alpha 1	Rn00578564_m1	71
	Itga10	integrin, alpha 10	Rn01533928_m1	73
	Itga11	integrin, alpha 11	Rn01421747_m1	54
	Itga2	integrin, alpha 2	Rn01489315_m1	52
	Itga2b	integrin, alpha 2B	Rn01456695_g1	108
	Itga3	integrin, alpha 3	Rn01751608_m1	61
	Itga4	integrin, alpha 4	Rn01512798_m1	97
	Itga5	integrin, alpha 5	Rn01761831_m1	91
	Itga6	integrin, alpha 6	Rn01512708_m1	53
	Itga7	integrin, alpha 7	Rn01529354_m1	56
	Itga8	integrin, alpha 8	Rn01487205_m1	78
	Itgal	integrin, alpha L	Rn01754645_m1	77
	Itgam	integrin, alpha M	Rn00709342_m1	76
	Itgav	integrin, alpha V	Rn01485633_m1	61
	Itgax	integrin, alpha X	Rn01511082_m1	64
	Itgb1	integrin, beta 1	Rn00566727_m1	81
	Itgb2	integrin, beta 2	Rn01427948_m1	142
	Itgb3	integrin, beta 3	Rn00596601_m1	66
	Itgb4	integrin, beta 4	Rn00566017_m1	66
	Itgb5	integrin, beta 5	Rn01439348_m1	63
	Itgb6	integrin, beta 6	Rn01747277_m1	82
	Limk1	LIM domain kinase 1	Rn00581900_m1	68
	Limk2	LIM domain kinase 2	Rn00577115_m1	60
Target genes	Llgl1	lethal giant larvae homolog 1	Rn00596096_m1	59
	Macf1	microtubule-actin crosslinking factor 1	Rn01516936_m1	56
	Map3k11	mitogen-activated protein kinase kinase kinase 11	Rn01502070_m1	149
	Map4	microtubule-associated protein 4	Rn01403680_m1	70
	Map6	microtubule-associated protein 6	Rn00567957_m1	95
	Mapk13	mitogen activated protein kinase 13	Rn00693138_m1	77
	Mapre1	microtubule-associated protein, RP/EB family, member 1	Rn00817431_g1	98
	Mapt	microtubule-associated protein tau	Rn01495715_m1	62
	Mark2	MAP/microtubule affinity-regulating kinase 2	Rn00433039_m1	68
	Mid1	midline 1	Rn00575975_m1	128
	Msn	moesin	Rn01424413_m1	58
	Mylk	myosin light chain kinase	Rn01439252_m1	72
	Mylk2	myosin light chain kinase 2	Rn00679009_m1	59
	Nck1	NCK adaptor protein 1	Rn01447186_m1	115
	Nck2	NCK adaptor protein 2	Rn01438228_m1	100
	Pak1	p21 protein (Cdc42/Rac)-activated kinase 1	Rn00664986_m1	98
	Pak2	p21 protein (Cdc42/Rac)-activated kinase 2	Rn00584005_m1	138
	Pak3	p21 protein (Cdc42/Rac)-activated kinase 3	Rn00693022_m1	72
	Pak4	p21 protein (Cdc42/Rac)-activated kinase 4	Rn01764387_m1	66
	Pak4	p21 protein (Cdc42/Rac)-activated kinase 4	Rn01764387_m1	66
	Parva	parvin, alpha	Rn00571958_m1	66
	Pdpk1	3-phosphoinositide dependent protein kinase-1	Rn00579366_m1	78
	Pfn2	profilin 2;profilin-2-like	Rn00450822_m1	104
	Phldb2	pleckstrin homology-like domain, family B, member 2	Rn01763372_m1	98
	Pip5k1c	phosphatidylinositol-4-phosphate 5-kinase, type I, gamma	Rn01408490_m1	61
	Plec	plectin	Rn00673737_m1	77
	Ppp1r12b	protein phosphatase 1, regulatory subunit 12B	Rn01490961_m1	67
	Ppp3ca	protein phosphatase 3, catalytic subunit, alpha isozyme	Rn00690508_m1	72
	Ppp3cb	protein phosphatase 3, catalytic subunit, beta isozyme	Rn00566864_m1	138
	Prkca	protein kinase C, alpha	Rn01496145_m1	74
	Prkcb	protein kinase C, beta	Rn00562312_m1	120
	Prkcg	protein kinase C, gamma	Rn00440861_m1	89
	Pten	phosphatase and tensin homolog	Rn00477208_m1	73
	Ptk2	protein tyrosine kinase 2	Rn01505115_m1	81
	Pxn	paxillin	Rn01499294_m1	71
	Rac1	ras-related C3 botulinum toxin substrate 1	Rn01412766_m1	86
	Rac2	ras-related C3 botulinum toxin substrate 2	Rn01504461_g1	57
	Racgap1	rac GTPase-activating protein 1	Rn01761773_m1	62
	Raf1	v-raf-leukemia viral oncogene 1	Rn00466507_m1	88
	Rap1a	RAP1A, member of RAS oncogene family	Rn01533681_g1	139
	Rap1b	RAP1B, member of RAS oncogene family	Rn01509461_gH	108
	Rapgef1	Rap guanine nucleotide exchange factor (GEF) 1	Rn01438718_m1	64
	Rasgrf1	RAS protein-specific guanine nucleotide-releasing factor 1	Rn01447219_m1	54
	Rhoa	ras homolog family member A	Rn04219609_m1	100
	Rock1	Rho-associated coiled-coil containing protein kinase 1	Rn00579490_m1	75
	Rock2	Rho-associated coiled-coil containing protein kinase 2	Rn00564633_m1	73
	Sh3rf1	SH3 domain containing ring finger 1	Rn01535121_m1	78
	Shc1	SHC (Src homology 2 domain containing) transforming protein 1	Rn01458709_g1	131
	Shc2	SHC (Src homology 2 domain containing) transforming protein 2	Rn01171467_m1	63
	Sos1	Son of sevenless homolog 1	Rn01473774_m1	141
	Sos2	Son of sevenless homolog 2	Rn01468822_m1	68
	Src	SRC proto-oncogene, non-receptor tyrosine kinase	Rn00583063_m1	75
	Ssh2	slingshot protein phosphatase 2	Rn01471631_m1	77
	Stmn1	stathmin 1	Rn00691285_g1	137
	Tiam1	T-cell lymphoma invasion and metastasis 1	Rn01477317_m1	61
	Tln1	talin 1	Rn01440247_m1	64
	Tns2	tensin 2	Rn01532783_m1	130
	Tns4	tensin 4	Rn01751085_m1	60
	Vasp	vasodilator-stimulated phosphoprotein	Rn01477032_m1	146
	Vav1	vav 1 guanine nucleotide exchange factor	Rn00562712_m1	69
	Vav2	vav 2 guanine nucleotide exchange factor	Rn01436349_m1	63
	Vcl	vinculin	Rn01755894_m1	63
	Was	Wiskott-Aldrich syndrome	Rn01528309_m1	82
	Wasf1	WAS protein family, member 1	Rn01754124_m1	72
	Wasl	Wiskott-Aldrich syndrome-like	Rn01501122_m1	63
	Zyx	zyxin	Rn01501253_m1	57
