# Supplementary material for: Unique osteogenic profile of bone marrow stem cells stimulated in perfusion bioreactor is Rho‐ROCK‐mediated contractility dependent
Source: Bioeng Transl Med. 2023 Mar 17;8(3):e10509. doi: 10.1002/btm2.10509 (PMC10189446; doi:10.1002/btm2.10509)
Supplement: Supplementary file 5 — Table S2: A set of genes and primers used for the assessment of osteogenic profile under perfusion [file BTM2-8-e10509-s004.pdf]

Table. S2 A set of genes and primers used for the assessment of osteogenic profile under perfusion

	Gene Symbol	Gene Name	TaqMan Assay ID	Amplicon Length (bp)
Target genes	Ahsg	alpha-2-HS-glycoprotein	Rn00563700_m1	113
	Alpl	alkaline phosphatase	Rn01516028_m1	68
	Anxa5	annexin A5	Rn00565571_m1	115
	Bglap	bone gamma-carboxyglutamate (gla) protein	Rn01455285_g1	81
	Bgn	biglycan	Rn00567229_m1	76
	Bmp1	bone morphogenetic protein 1	Rn01466016_m1	75
	Bmp2	bone morphogenetic protein 2	Rn00567818_m1	126
	Bmp3	bone morphogenetic protein 3	Rn00690955_mH	60
	Bmp4	bone morphogenetic protein 4	Rn00432087_m1	61
	Bmp5	bone morphogenetic protein 5	Rn01447676_m1	78
	Bmp6	bone morphogenetic protein 6	Rn00432095_m1	122
	Bmp7	bone morphogenetic protein 7	Rn01528889_m1	65
	Bmpr1a	bone morphogenetic protein receptor, type IA	Rn01450292_g1	82
	Bmpr1b	bone morphogenetic protein receptor, type IB	Rn01748421_m1	67
	Cdh11	cadherin 11	Rn01536913_m1	61
	Col10a1	collagen, type X, alpha 1	Rn01408030_m1	80
	Col11a1	collagen, type XI, alpha 1	Rn01523309_m1	54
	Col12a1	collagen, type XII, alpha 1	Rn01521220_m1	57
	Col14a1	collagen, type XIV, alpha 1	Rn01502357_m1	57
	Col1a1	collagen, type I, alpha 1	Rn01463848_m1	115
	Col1a2	collagen, type I, alpha 2	Rn01526721_m1	96
	Col2a1	collagen, type II, alpha 1	Rn01637087_m1	97
	Col3a1	collagen, type III, alpha 1	Rn01437681_m1	71
	Col4a1	collagen, type IV, alpha 1	Rn01482927_m1	69
	Col4a2	collagen, type IV, alpha 2	Rn01482133_m1	53
	Col5a1	collagen, type V, alpha 1	Rn00593170_m1	61
	Col6a1	collagen, type VI, alpha 1	Rn01429556_m1	67
	Col6a2	collagen, type VI, alpha 2	Rn01429497_m1	77
	Col7a1	collagen, type VII, alpha 1	Rn01544829_m1	60
	Comp	cartilage oligomeric matrix protein	Rn00563255_m1	87
	Csf2	colony stimulating factor 2	Rn01456851_m1	127
	Csf3	colony stimulating factor 3	Rn00567344_m1	95
	Ctsk	cathepsin K	Rn00580723_m1	69
	Cyr61	cysteine-rich, angiogenic inducer, 61	Rn00580055_m1	113
	Dmp1	dentin matrix acidic phosphoprotein 1	Rn01450122_m1	77
	Egf	epidermal growth factor	Rn00563336_m1	93
	Fgf1	fibroblast growth factor 1	Rn00689153_m1	78
	Fgf2	fibroblast growth factor 2	Rn00570809_m1	63
	Fgf3	fibroblast growth factor 3	Rn00590754_m1	60
	Fgfr1	Fibroblast growth factor receptor 1	Rn00577234_m1	78
	Fgfr2	fibroblast growth factor receptor 2	Rn01269940_m1	87
	Flt1	FMS-related tyrosine kinase 1	Rn00570815_m1	60
	Fn1	fibronectin 1	Rn00569575_m1	97
	Gdf10	growth differentiation factor 10	Rn00666937_m1	105
	Ibsp	integrin-binding sialoprotein	Rn00561414_m1	138
	Icam1	intercellular adhesion molecule 1	Rn00564227_m1	61
	Igf1	insulin-like growth factor 1	Rn00710306_m1	69
	Igf1r	insulin-like growth factor 1 receptor	Rn00583837_m1	58
	Itga2	integrin, alpha 2	Rn01489315_m1	52
	Itga3	integrin, alpha 3	Rn01751608_m1	61
	Itgb1	integrin, beta 1	Rn00566727_m1	81
	Mmp10	matrix metallopeptidase 10	Rn00591678_m1	79
	Mmp2	matrix metallopeptidase 2	Rn01538170_m1	63
	Mmp8	matrix metallopeptidase 8	Rn00573646_m1	92
	Mmp9	matrix metallopeptidase 9	Rn00579162_m1	72
	Msx1	msh homeobox 1	Rn00667535_m1	83
	Nfkb1	nuclear Factor Kappa B Subunit 1	Rn01399583_m1	63
	Pdgfa	platelet-derived growth factor alpha polypeptide	Rn00709363_m1	62
	Phex	phosphate regulating endopeptidase homolog, X-linked	Rn00448130_m1	95
	Runx2	runt-related transcription factor 2	Rn01512298_m1	86
	Scarb1	scavenger receptor class B, member 1	Rn00580588_m1	75
	Serpinh1	Serpin Family H Member 1	Rn00567777_m1	76
	Smad1	SMAD family member 1	Rn00565555_m1	79
	Smad2	SMAD family member 2	Rn00569900_m1	67
	Smad3	SMAD family member 3	Rn00565331_m1	94
	Smad4	SMAD family member 4	Rn00570593_m1	101
	Sost	sclerostin	Rn00577971_m1	81
	Sox9	SRY (sex determining region Y)-box 9	Rn01751069_mH	60
	Sp7	Sp7 transcription factor	Rn01761789_m1	107
	Spp1	secreted phosphoprotein 1	Rn00681031_m1	73
	Tfip11	tuftelin interacting protein 11	Rn01481123_m1	125
	Tgfb1	transforming growth factor, beta 1	Rn00572010_m1	65
	Tgfb2	transforming growth factor, beta 2	Rn00579674_m1	95
	Tgfb3	transforming growth factor, beta 3	Rn00565937_m1	61
	Tgfbr1	transforming growth factor, beta receptor 1	Rn00562811_m1	86
	Tgfbr2	transforming growth factor, beta receptor 2	Rn00579682_m1	74
	Tgfbr3	transforming growth factor, beta receptor 3	Rn00568482_m1	89
	Tnf	tumor necrosis factor	Rn00562055_m1	82
	Tuft1	tuftelin 1	Rn01762781_m1	68
	Twist1	twist family bHLH transcription factor 1	Rn00585470_s1	71
	Vcam1	vascular cell adhesion molecule 1	Rn00563627_m1	90
	Vdr	vitamin D receptor	Rn00690616_m1	63
	Vegfa	vascular endothelial growth factor A	Rn01511601_m1	69
	Vegfb	vascular endothelial growth factor B	Rn01454585_g1	68
