# Supplementary material for: Unique osteogenic profile of bone marrow stem cells stimulated in perfusion bioreactor is Rho‐ROCK‐mediated contractility dependent
Source: Bioeng Transl Med. 2023 Mar 17;8(3):e10509. doi: 10.1002/btm2.10509 (PMC10189446; doi:10.1002/btm2.10509)
Supplement: Supplementary file 6 — Table S3: A set of genes and primers used as an endogeneous control [file BTM2-8-e10509-s006.pdf]

Table. S3 A set of genes and primers used as an endogeneous control

	Gene Symbol	Gene Name	TaqMan Assay ID	Amplicon Length (bp)
Endogenous control	18S	Eukaryotic 18S rRNA	Hs99999901_s1	187
	B2m	beta-2 microglobulin	Rn00560865_m1	58
	Gapdh	glyceraldehyde-3-phosphate dehydrogenase	Rn99999916_s1	87
	Gusb	glucuronidase, beta	Rn00566655_m1	63
	Hmbs	hydroxymethylbilane synthase	Rn00565886_m1	99
	Hprt1	hypoxanthine phosphoribosyltransferase 1	Rn01527840_m1	64
	Pgk1	phosphoglycerate kinase 1	Rn00821429_g1	84
	Rplp0	ribosomal protein, large, P0	Rn00821065_g1	97
	Rplp2	ribosomal protein, large P2	Rn01479927_g1	130
